# Electrolytic Seawater Mineralization and the Mass Balances That Demonstrate Carbon Dioxide Removal

**DOI:** 10.1021/acsestengg.3c00004

**Published:** 2023-04-27

**Authors:** Erika Callagon La Plante, Xin Chen, Steven Bustillos, Arnaud Bouissonnie, Thomas Traynor, David Jassby, Lorenzo Corsini, Dante A. Simonetti, Gaurav N. Sant

**Affiliations:** † Department of Materials Science and Engineering, University of Texas at Arlington, Arlington, Texas 76019, United States; ‡ Institute for Carbon Management, University of California, Los Angeles, Los Angeles, California 90024, United States; § Center for Advanced Construction Materials, University of Texas at Arlington, Arlington, Texas 76019, United States; ∥ Equatic Inc., Los Angeles, California 90024, United States; ⊥ Department of Civil and Environmental Engineering, University of California, Los Angeles, Los Angeles, California 90024, United States; # Department of Chemical and Biomolecular Engineering, University of California, Los Angeles, Los Angeles, California 90024, United States; ∇ California Nanosystems Institute, University of California, Los Angeles, Los Angeles, California 90024, United States; ○ Department of Materials Science and Engineering, University of California, Los Angeles, Los Angeles, California 90024, United States

**Keywords:** Carbon dioxide mineralization, calcium carbonate, hydrated magnesium carbonate, brucite, electrolysis

## Abstract

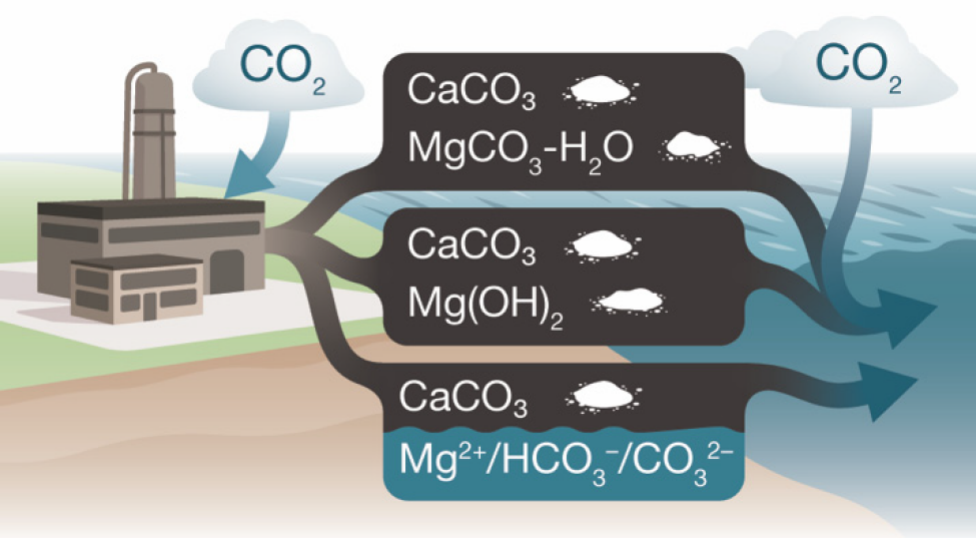

We present the mass balances associated with carbon dioxide (CO_2_) removal (CDR) using seawater as both the source of reactants and as the reaction medium via electrolysis following the “*Equatic*” (*formerly known as “SeaChange*”) process. This process, extensively detailed in La PlanteE.C.; ACS Sustain. Chem. Eng.2021, 9, ( (3), ), 1073–1089, involves the application of an electric overpotential that splits water to form H^+^ and OH^–^ ions, producing acidity and alkalinity, i.e., in addition to gaseous coproducts, at the anode and cathode, respectively. The alkalinity that results, i.e., via the “continuous electrolytic pH pump” results in the instantaneous precipitation of calcium carbonate (CaCO_3_), hydrated magnesium carbonates (e.g., nesquehonite: MgCO_3_·3H_2_O, hydromagnesite: Mg_5_(CO_3_)_4_(OH)_2_·4H_2_O, etc.), and/or magnesium hydroxide (Mg(OH)_2_) depending on the CO_3_^2–^ ion-activity in solution. This results in the trapping and, hence, durable and permanent (at least ∼10 000–100 000 years) immobilization of CO_2_ that was originally dissolved in water, and that is additionally drawn down from the atmosphere within: (a) mineral carbonates, and/or (b) as solvated bicarbonate (HCO_3_^–^) and carbonate (CO_3_^2–^) ions (i.e., due to the absorption of atmospheric CO_2_ into seawater having enhanced alkalinity). Taken together, these actions result in the net removal of ∼4.6 kg of CO_2_ per m^3^ of seawater catholyte processed. Geochemical simulations quantify the extents of net CO_2_ removal including the dependencies on the process configuration. It is furthermore indicated that the efficiency of realkalinization of the acidic anolyte using alkaline solids depends on their acid neutralization capacity and dissolution reactivity. We also assess changes in seawater chemistry resulting from Mg(OH)_2_ dissolution with emphasis on the change in seawater alkalinity and saturation state. Overall, this analysis provides direct quantifications of the ability of the *Equatic* process to serve as a means for technological CDR to mitigate the worst effects of accelerating climate change.

## Introduction and Background

The trapping of carbon dioxide (CO_2_) as an aqueous (bi)carbonate ion (e.g., HCO_3_^–^, CO_3_^2–^) or as a mineral solid (“mineralization”) is attractive because of favorable thermodynamics and the safety and permanence of storage.^2−4^ Furthermore, mineralization is a cost-effective pathway for CO_2_ sequestration/removal (CDR),^5,6^ which, at steady state, is estimated to cost <$100 per tonne (t) of CO_2_.^2^ During CO_2_ mineralization, the release of Ca and Mg from the precursor solids is rate-limiting, unless mass transport is hindered, which is seldom the case.^7^ Thus, providing presolubilized cations that can readily react with CO_2_ enormously facilitates mineralization rates and extents. Seawater is a vast reservoir of divalent cations (Ca^2+^, Mg^2+^) and dissolved CO_2_ that can form sparingly soluble carbonates (and/or hydroxides). Long-term (millions of years) storage of CO_2_ on Earth occurs by mineralization through the formation of calcite (CaCO_3_) and aragonite (CaCO_3_). But, over the short term, the abiotic precipitation of Ca and Mg carbonates from seawater is kinetically inhibited, as implied by the supersaturation of oceans with respect to these minerals.

The oceans absorb and immobilize atmospherically derived CO_2_ in the form of dissolved carbonate species (i.e., predominantly HCO_3_^–^ at a prevailing pH of ∼8.1). Such aqueous immobilization is highly durable, although less so than mineral carbonate formation (i.e., which has a stability of up to billions of years),^8,9^ and presents a lower bound of stability in excess of 10 000 years.^10,11^ As a result, 25% of all anthropogenic CO_2_ emissions (∼9 gigatonnes, Gt) are removed from the atmosphere by the oceans annually.^12^ But, as a function of their prevailing chemistry^1^ and ongoing ocean acidification, the capacity of the oceans to absorb additional CO_2_ (i.e., annually and per unit of seawater) is capped, unless prevalent CO_2_ were to be removed. Toward this end, i.e., to remove CO_2_ from the oceans and to expand the capacity of seawater to absorb additional CO_2_, several electrochemical processes have been proposed, which focus on increasing ocean alkalinity via the: (a) production of OH^–^ from seawater (and the utilization of the HCl coproduct to accelerate silicate weathering),^13^ (b) using hard water and ion-exchange membranes,^14,15^ or (c) utilizing pH swing processes to extract and capture CO_2_.^16,17^

Recently, in La Plante et al., we proposed an approach to rapidly precipitate Ca and Mg carbonates and hydroxides from seawater to achieve CDR.^1^ This *Equatic* process electrolytically forces mineral carbonate precipitation thereby consuming prevalent CO_2_ that is dissolved in seawater by locking it within carbonate minerals and, simultaneously, producing alkaline mineral hydroxides that, when dissolved in seawater, enable the drawdown of atmospheric CO_2_ into the seawater ensuring net CO_2_ removal.^1^ As such, previously, in La Plante et al., we have carefully examined and assessed the *Equatic* approach via detailed evaluations of energy demands, process cost, implementation schemes, and the achievable scale of carbon removal.^1^ Therefore, in the current paper we particularly (only) focus on describing the geochemical basis and the CO_2_ (mass) balances of the *Equatic* process. Two scenarios are presented: (1) the precipitation of calcium carbonate and magnesium hydroxide (Mg(OH)_2_: brucite), with Mg(OH)_2_ dispersed as solids or dissolved in seawater and pre-equilibrated with CO_2_ under dilute conditions, and (2) the precipitation of calcium and hydrated magnesium carbonates, i.e., when Mg(OH)_2_ is carbonated under nondilute conditions. Special focus is paid to offer detailed quantifications of carbon mass balances based on equilibrium calculations. The analysis, therefore, offers a quantitative basis for assessing the CDR potential of the technology and for developing a robust measurement, reporting, and verification (MRV) strategy. This manuscript provides limited discussion around the full life cycle of the process, including electrolyzer materials and systems, balance-of-plant equipment, operational considerations, etc. These aspects represent ongoing work that will be addressed in future publications. Taken together, these efforts contribute to the mitigation of ongoing climate change, which poses enormously negative effects on ecosystems and people’s quality of life.^18^

## Analysis Methods

We use PHREEQC^19^ to carry out detailed geochemical simulations. The llnl.dat database was used, which is appropriate for ionic strengths up to seawater salinity (up to ∼1 molal (mol/kg); for comparison, seawater’s ionic strength is 0.7 molal) and which explicitly considers metal complexation with carbon. This database uses the Debye–Hückel model with the B-dot equation and includes an explicit expression for the activity coefficient of aqueous carbon dioxide (CO_2(aq)_) as a function of temperature and ionic strength.^19^ The seawater composition used is based on Millero et al. (2008) (Table 1),^20^ adjusted to pCO_2_ (in atm) = −3.38 (420 ppm)^21^ by charge balancing for the presence of inorganic C (carbon) species. Similar results are obtained using the pitzer.dat database, which is suitable for solutions having higher ionic strengths (>1 molal) but does not contain thermodynamic data for hydromagnesite and cannot be extended above 25 °C. For example, the pH obtained after equilibration at 420 ppm of CO_2_ is 8.258 when using the pitzer.dat database and 8.170 when using the llnl.dat database (Table 1). The Saturation Index (SI) is defined as log Ω, where the saturation ratio, Ω = *Q*/*K*_sp_, and *Q* is the ion activity product and *K*_sp_ is the solubility product with respect to a given mineral. The saturation indices and ratios with respect to relevant Mg- and Ca-based minerals in seawater are shown in Table 2. In brief, seawater is supersaturated with respect to aragonite, calcite, dolomite, and magnesite and undersaturated with respect to the hydrated magnesium carbonates and brucite. All the calculations assume thermodynamic equilibrium for *T* = 25 °C, *p* = 1 bar (1 atm).

**Table 1:** Composition of Seawater Used in the Analysis

**Species**	**Molality (***m***,** mol/kg)**based on Reference Composition**^20^	**Molality (***m***,**mol/kg) **after equilibration at 420 ppm of CO**_**2(g)**_
Na^+^	0.4860597	0.4860597
Mg^2+^	0.0547421	0.0547421
Ca^2+^	0.0106568	0.0106568
K^+^	0.0105797	0.0105797
Sr^2+^	0.0000940	0.0000940
Cl^–^	0.5657647	0.5657647
SO_4_^2–^	0.0292643	0.0292643
HCO_3_^–^	0.0017803	0.0021002
Br^–^	0.0008728	0.0008728
CO_3_^2–^	0.0002477	0.0000312
F^–^	0.0000708	0.0000708
B [B(OH)_4_^–^, B(OH)_3_]	0.0004303	0.0004303
H_2_CO_3_*	0.0000100	0.0000124
ΣCO_2_	2.038 mmol/kg	2.141 mmol/kg
pH	8.352	8.170
pCO_2_ (in atm)	–3.78	–3.38

**Table 2:** Saturation Indices and Ratios of Different Mineral Solids in Seawater at 25 °C and pCO_2_ = −3.38 atm

**Phase**	**Composition**	**Saturation Index, SI**	**Saturation Ratio, Ω**
Aragonite	CaCO_3_	0.52	3.311
Artinite	Mg_2_CO_3_(OH)_2_·6H_2_O	–1.97	0.011
Brucite	Mg(OH)_2_	–1.84	0.014
Calcite	CaCO_3_	0.67	4.677
Dolomite	CaMg(CO_3_)_2_	3.26	1819.7
Huntite	CaMg_3_(CO_3_)_4_	1.99	97.72
Hydromagnesite	Mg_5_(CO_3_)_4_(OH)_2_·4H_2_O	–3.38	0.0004
Lansfordite	MgCO_3_·5H_2_O	–1.64	0.023
Magnesite	MgCO_3_	0.97	9.333
Nesquehonite	MgCO_3_·3H_2_O	–2.07	0.009

The CO_2_ content (i.e., storage capacity) of seawater is dependent on its alkalinity. The total alkalinity (*A*_T_, mg/L) of seawater is given by
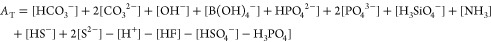
or equivalently
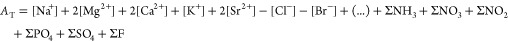
where (···) represents minor conservative species, ∑NH_3_ = NH_3_ + NH_4_^+^, ∑NO_3_ = NO_3_^–^ + HNO_3_, ∑NO_2_ = NO_2_^–^ + HNO_2_, ∑PO_4_ = H_3_PO_4_ + H_2_PO_4_^–^ + HPO_4_^2–^ + PO_4_^3–^, ∑SO_4_ = H_2_SO_4_ + HSO_4_^–^ + SO_4_^2–^, and ∑F = HF + F^–^.^22−24^

## Results and Discussion

### Carbon Dioxide Dissolution in Seawater

The equilibrium of gas-phase CO_2_ with seawater is described in detail elsewhere.^25−27^ Briefly, the dissolved CO_2_ content in seawater is controlled by its pH, the atmospheric partial pressure of CO_2_ (pCO_2_), and the temperature as described by Henry’s law. The relative concentrations of HCO_3_^–^, CO_3_^2–^, and H_2_CO_3_*, which denotes the sum of H_2_CO_3_ (carbonic acid) and aqueous CO_2_, [HCO_3_^–^], [CO_3_^2–^], and [H_2_CO_3_*], are determined via the equilibrium constants *K*_H_, *K*_1_, and *K*_2_ (see eqs 1–3), which are functions of the temperature and the salinity of the water.^26^ In PHREEQC, there is a single equilibrium constant that can be applied across all concentrations because the underlying (law of) mass action equations are written in terms of discrete ion activities. While small differences are indeed possible for the absolute numerical values of temperature/salinity-dependent equilibrium constants when solved using eqs 1–3, we chose the approach embedded in PHREEQC because it allows for customized modeling of numerous scenarios while accounting for the effects of solution nonideality (i.e., wherein activity and concentration are inequivalent) explicitly.
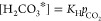
1
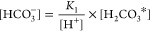
2
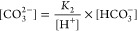
3

**Figure 1:**
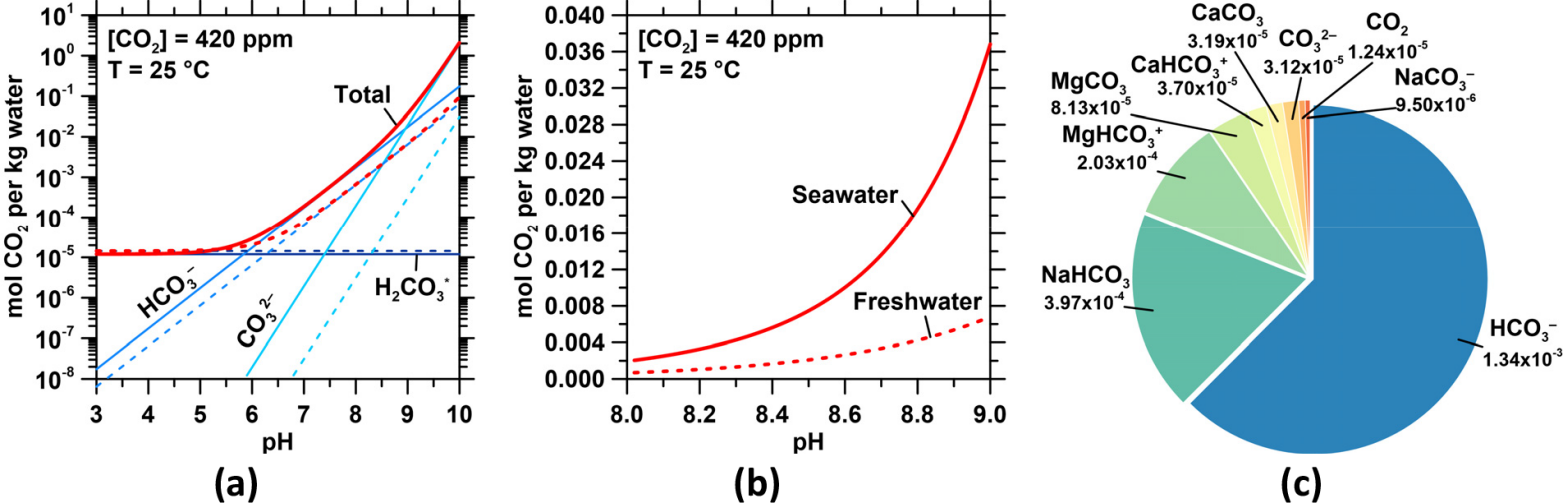
(a) The concentration and speciation of CO_2_ in seawater (solid curves) and freshwater (dashed curves) in equilibrium with an ambient atmosphere containing 420 ppm of CO_2_ (0.042 vol % CO_2_). The total dissolved CO_2_ is the sum of the concentrations of HCO_3_^–^, CO_3_^2–^, and H_2_CO_3_*. H_2_CO_3_* represents the sum of CO_2(aq)_ and true carbonic acid (H_2_CO_3_). The speciation of CO_2_ is calculated using equilibrium constants that vary with temperature and salinity.^26^ (b) The detail of (a) for 8 ≤ pH ≤ 9, showing the far greater solubility of CO_2_ in seawater than freshwater. (c) The different aqueous species of DIC, including complexes with dissolved cations in seawater, and their relative amounts. The concentrations for each species are given in mol/kg (molal basis).

Here, eq 1 is Henry’s law, where *K*_H_ is the Henry’s law constant (0.03428 mol/L/atm for freshwater (0 per mil, ‰) and 0.02858 for seawater (35 ‰)), and *p* is the partial pressure in atm (i.e., 420 ppm is equivalent to 0.00042 atm). The equilibrium constant *K*_1_ is taken as 4.498 × 10^–7^ mol/kg for freshwater and 14.52 × 10^–7^ mol/kg for seawater, whereas *K*_2_ is taken as 0.479 × 10^–10^ mol/kg for freshwater and 11.12 × 10^–10^ mol/kg for seawater.^26,28^ Refinements to such equilibrium constants have been the subject of past studies^25,29−31^ but are beyond the scope of the current work. The concentration of aqueous H^+^ is equivalent to 10^–pH^ (where the ionic product of water *K*_w_ = 10^–14^). The speciation of CO_2_ and the relative abundances of HCO_3_^–^ and CO_3_^2–^ show a strong dependence on the pH (Figure 1a). On the other hand, H_2_CO_3_* is controlled by pCO_2_ and is independent of pH. The equilibrium between H_2_CO_3_ and CO_2(aq)_ is given by [H_2_CO_3_] = *K*_0_[CO_2(aq)_], where p*K*_0_ = 2.97, indicating that CO_2(aq)_ is ∼1000 times more abundant than H_2_CO_3_.^32^ Thus, the total dissolved CO_2_ (total dissolved inorganic carbon: DIC, ΣCO_2_) is given by the sum of the different carbon species and is equal to ∑CO_2_ = [H_2_CO_3_*] + [HCO_3_^–^] + [CO_3_^2–^]. At pH 8.1 and 420 ppm of CO_2_, the total dissolved CO_2_ concentrations in freshwater and seawater based on this analysis are 0.847 and 2.557 mmol CO_2_/kg water, in reasonable agreement with Table 1 albeit with a discrepancy that is caused by differences in the equilibrium constants that are used.^33^ Importantly, since we are assuming cation-limited reactions, the calculated value of total dissolved CO_2_ is not used in the carbon mass balances in this paper. Furthermore, as relevant, the discussion below is based on PHREEQC calculations that resulted in dissolved CO_2_ concentrations lower than the calculations based on eqs 1–3. Notably, the equilibrium ΣCO_2_ in seawater is greater than that in freshwater because of the higher ionic strength of seawater that results in the speciation of CO_2_ into HCO_3_^–^ and CO_3_^2–^ by complexation of the bicarbonate and carbonate ions with cations such as Na^+^, Mg^2+^, and Ca^2+^ (see Figure 1b,c).

### Electrolytic Carbon Removal

The *Equatic* process consists of the following steps.

Step 1) The precipitation of brucite (Mg(OH)_2_) and aragonite (CaCO_3_) at the catholyte, consuming 100% of initial [Mg] and 20% of initial [Ca] in seawater.

Step 2) Catholyte processing, either Step 2a or Step 2b.

Step 2a) The solids are separated from the catholyte effluent (Table 3). Residual Ca^2+^ in the catholyte precipitates as CaCO_3_ in a carbonation reactor, while Mg(OH)_2_ solids are (a) discharged into the ocean (**Case 1**) or (b) pre-equilibrated with seawater and carbonated inside the plant’s battery limits under dilute (mass) conditions (<∼0.002 mol Mg(OH)_2_/kg seawater, see Figure 7d) (**Case 2a**).

**Table 3:** Representative Steady-State Composition of the Anolyte and Catholyte Effluent Exiting the Electrolyzer (see Figure 2) at Ambient (*p*,*T*) and in Equilibrium with Air

**Species**	**Anolyte Molality**(mol/kg)	**Catholyte Molality**(mol/kg)
Na^+^	0.4110597	0.6228000
Mg^2+^	0.0547421	0
Ca^2+^	0.0106568	0.0083568
K^+^	0.0105797	0.0105797
Sr^2+^	0.0000940	0.0000940
Cl^–^	0.6367647	0.5680000
SO_4_^2–^	0.0292643	0.0292643
HCO_3_^–^	0	0
Br^–^	0.0008728	0.0008728
CO_3_^2–^	0	0
F^–^	0.0000708	0.0000708
B [B(OH)_4_^–^, B(OH)_3_]	0.0004303	0.0004303
H_2_CO_3_*	0.0000164	0
ΣCO_2_	0.0000164	0
CaCO_3_ (s)	0	0.002
Mg(OH)_2_ (s)	0	0.055
pH	1.023	12.200
pCO_2_	–3.38	-

Step 2b) The catholyte effluent containing both solids and ions is carbonated inside plant limits, resulting in the dissolution of Mg(OH)_2_ and equilibration with a CO_2_-enriched vapor under nondilute (mass) conditions to produce hydrated Mg carbonates, while the residual Ca^2+^ in solution precipitates as CaCO_3_ (**Case 2b**).

Step 3) The realkalinization of the anolyte stream to neutralize its acidity and replenish divalent cations that are consumed (and do not redissolve) during mineral precipitation.

Step 4) The discharge of the processed anolyte and catholyte streams back into the ocean.^1^

Ultimately, the process traps CO_2_ as (a) dissolved (i.e., aqueous HCO_3_^–^ and CO_3_^2–^) species stabilized via the redissolution of Mg(OH)_2_) and/or (b) solid (e.g., CaCO_3_, a mineral carbonate) forms. This manner of CDR is represented by two limiting cases: (**Cases 1, 2a**) CaCO_3_ + Mg(OH)_2_ (i.e., 89 mass % aqueous, 11 mass % solid CO_2_ immobilization) and (**Case 2b**) CaCO_3_ + Mg–CO_3_ hydrates (i.e., 100 mass % solid CO_2_ immobilization). The CaCO_3_ solids produced via this process can be discharged back into the ocean, where they will remain stable because of their native prevalence and persistence (e.g., seashells in the ocean) and seawater’s supersaturation with respect to the mineral carbonates (Table 2), or they will be beneficially utilized, e.g., as sand in concrete, or as a carbon-neutral feedstock to produce cement. Obviously, if hydrated carbonate phases including nesquehonite (MgCO_3_·3H_2_O), lansfordite (MgCO_3_·5H_2_O), hydromagnesite (Mg_5_(CO_3_)_4_(OH)_2_·4H_2_O), and dypingite (Mg_5_(CO_3_)_4_(OH)_2_·5H_2_O) form, alternative disposal strategies (e.g., on land) will be needed due to the tendency of these solids to dissolve if they were to be discharged into the ocean (Table 2).

From stoichiometry, the formation of 1 mol of CaCO_3_ or Mg–CO_3_ hydrates (e.g., nesquehonite: MgCO_3_·3H_2_O) captures 1 mol of CO_2_, while requiring 2 mol of OH^–^. For comparison, only 1.2 mol of OH^–^ are required per mole of CO_2_ stored as dissolved (bicarbonate: HCO_3_^–^ and carbonate: CO_3_^2–^) ions (Figures 1 and 7).^1,10^ This implies that, per unit of alkalinity, it is more chemically and energy efficient to immobilize CO_2_ in the form of dissolved aqueous carbonates, i.e., rather than mineral carbonate species. The *Equatic* process is based on the electrolysis of seawater. Such electrochemical stimulation of seawater implies the formation of alkalinity (OH^–^) at the cathode and acidity (H^+^) at the anode. In addition, gas-phase coproducts evolve, including hydrogen (H_2(g)_) at the cathode and oxygen (O_2(g)_) and chlorine (Cl_2(g)_) at the anode. These gas evolutions are described by the hydrogen evolution reaction (HER), oxygen evolution reaction (OER), and chlorine evolution reaction (ClER), respectively. During seawater electrolysis, unless an oxygen-selective anode is used, ClER is the predominant reaction at the anode because its 2e^–^ basis (i.e., as compared to the 4e^–^ basis of OER) makes its formation more kinetically favorable as compared to oxygen evolution, which is thermodynamically favored.

**Figure 2:**
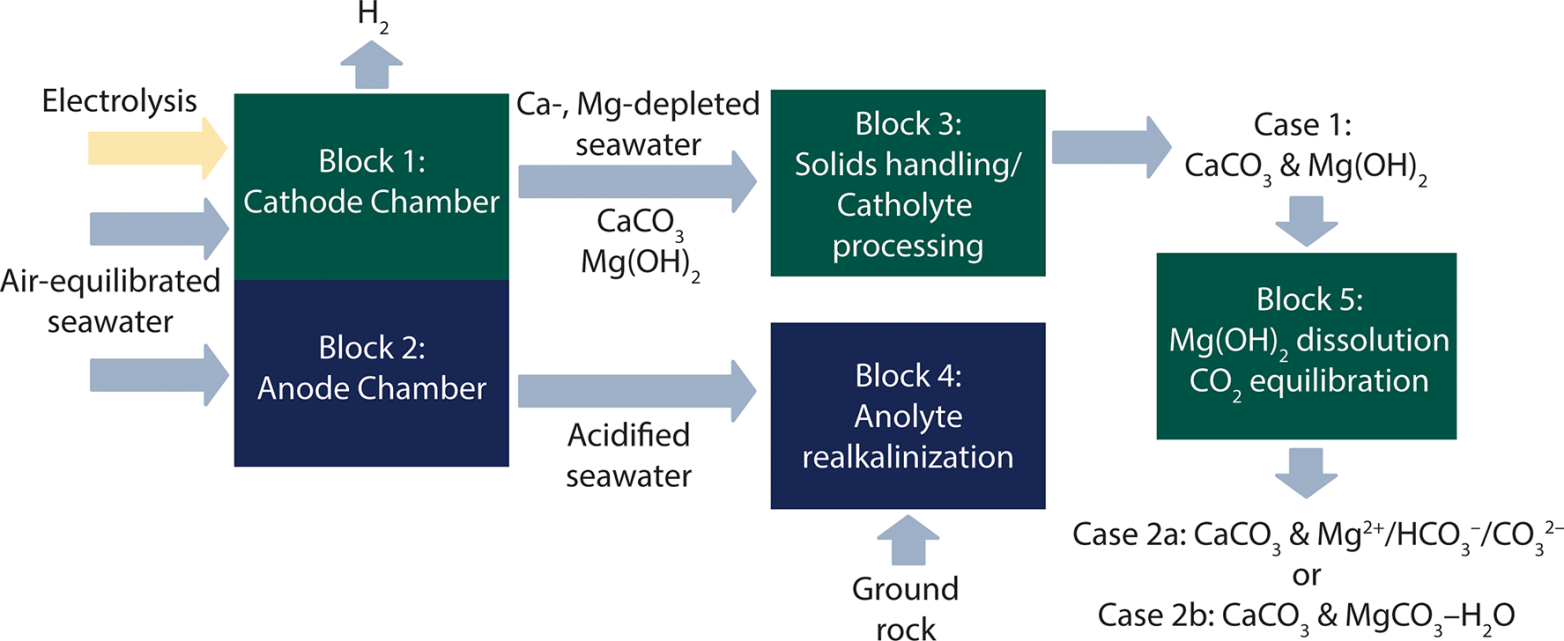
A schematic of the *Equatic* process showing major inlet and outlet feeds of the primary steps for CO_2_ removal associated with the formation of: carbonate solids and (aqueous) dissolved CO_2_ (**Cases 1, 2a**) and carbonate solids only (**Case 2b**). The major energy inputs include electrolysis, water processing and pumping, and rock grinding.^1^

The *Equatic* process’s mass balances can be examined for a system that removes 1 t of CO_2_ per day (TPD). For **Cases 1** and **2a**, this system requires the processing of ∼220 m^3^ per day of seawater in the catholyte to yield 235 kg of CaCO_3_ and 702 kg of Mg(OH)_2_ (i.e., if the solids were suspended in the solution this translates to ∼0.4 mass % solids, corresponding to a dilute system) while assuming a CO_2_ removal efficiency of 1.7 mol of CO_2_ per mol of Mg(OH)_2_ (see below). In addition, ∼29 kg of H_2(g)_, ∼46 kg of O_2(g)_, and ∼818 kg of chlorine (Cl_2(g)_, HClO, and ClO^–^) are produced when using an anode that is not OER-selective (e.g., platinum). The amount of free chlorine generated can be reduced greatly by the use of oxygen-selective anodes, without affecting the overpotential, and achieving >98 mass % selectivity of the OER as compared to ClER—a fast maturing effort that addresses obvious issues related to toxicity, handling, and atmospheric release of chlorine and chlorine derivatives. For these considerations independent of whether chlorine is evolved (and scrubbed) or suppressed, **Cases 1** and **2a** yields net 4.6 kg of CO_2_ removal per m^3^ of seawater processed as catholyte. Herein, CO_2_ removal via the alkalinity enhancement enabled by the dissolution of brucite (Mg(OH)_2_) can be effected in the ocean, i.e., following the oceanic discharge of the brucite, or within a captive carbonation/aeration reactor wherein air is sparged into the solution and CO_2_ absorption and bicarbonate/carbonate ion formation occur following Henry’s Law (see further discussion below; and Figure 2).

Alternatively, **Case 2b** requires 348 m^3^ per day of seawater in the catholyte and produces 371 kg of CaCO_3_ and 2635 kg of MgCO_3_·3H_2_O or 8909 kg of Mg_5_(CO_3_)_4_(OH)_2_·4H_2_O, depending on which hydrated magnesium carbonate phase forms (i.e., if the solids were suspended in the solution this translates to ∼0.9 to 2.7 mass % solids, corresponding to a dilute system). In addition, ∼46 kg of H_2(g)_, ∼73 kg of O_2(g)_, and ∼1295 kg of free chlorine (Cl_2(g)_, HClO, and ClO^–^) are produced when using a platinum-based anode that is not OER-selective. Thus, **Case 2b** yields net 2.9 kg of CO_2_ removal per m^3^ of seawater processed as catholyte. The carbonation of the catholyte to produce Mg–CO_3_ hydrates (Step 2b) implies the bubbling of a CO_2_-enriched vapor into the catholyte effluent, e.g., as sourced from air, a fractional direct air capture (DAC) system, or a CO_2_-enriched flue gas emissions stream. The mass and energy inputs relevant to **Cases 1**, **2a**, and **2b** are shown schematically in Figure 2 and are described elsewhere.^1^

#### Precipitation of Calcium Carbonate and Magnesium Hydroxide

The ocean is supersaturated with respect to aragonite by a factor of at least 2–3 (Table 1), implying that the kinetic inhibition of precipitation is operative.^34^ With decreasing Ω, the time elapsed before the onset of mineral precipitation increases gradually at Ω > 3 and then sharply at Ω ≈ 3, implying seawater stability at Ω < 3.^35^ This kinetic inhibition of precipitation is caused by dissolved organic matter,^36,37^ phosphate ions,^38^ magnesium ions,^39^ and sulfate ions.^40^ To overcome the kinetic hindrance to precipitation we alkalinize the electrolyte such that, e.g., at pH 10–12, in the vicinity of the cathode we ensure Ω > 1400 (at pH 10) for calcite, and Ω > 7 (at pH 10) for brucite for seawater in equilibrium with air: i.e., for saturation ratios which are more than sufficient to overcome the thermodynamic and kinetic barriers to mineral precipitation.

**Figure 3:**
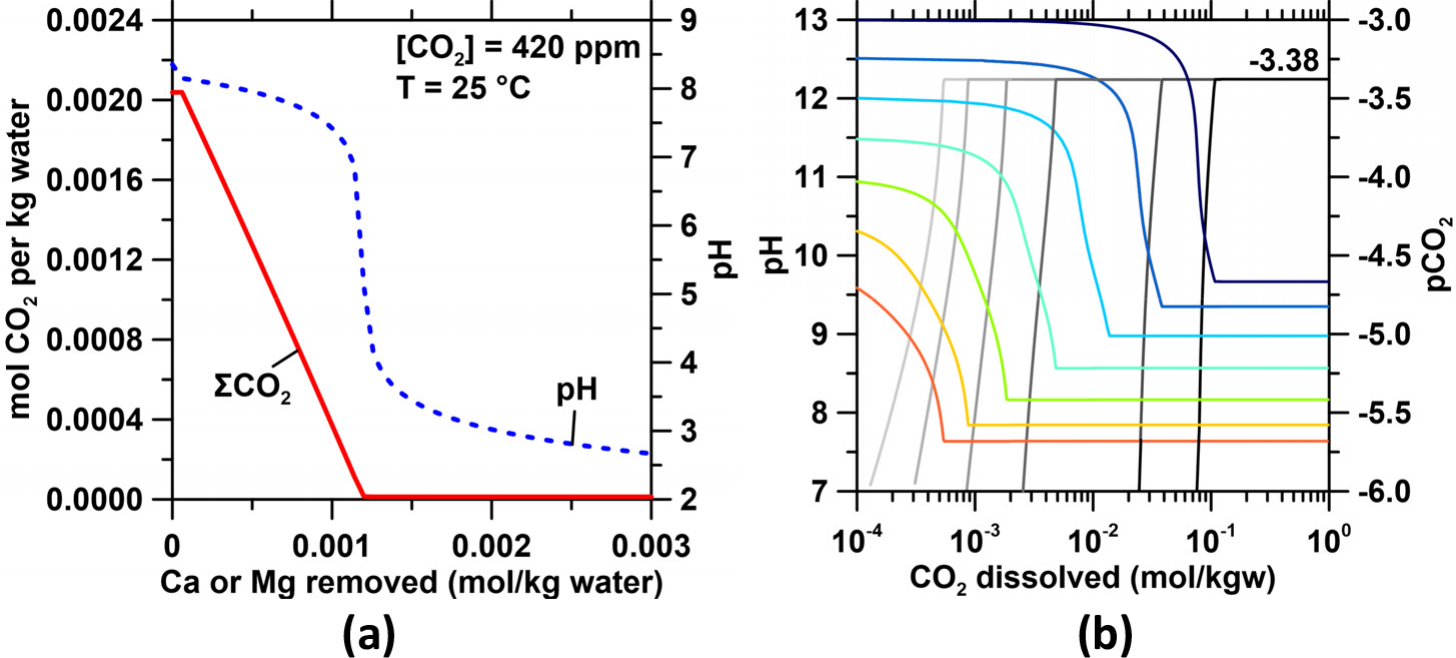
(a) The evolution of total dissolved CO_2_ (ΣCO_2_) and the pH of seawater with increasing Ca^2+^ and Mg^2+^ precipitation as CaCO_3_, Mg–CO_3_ hydrates, and/or Mg(OH)_2_. (b) The equilibration with air of the catholyte effluent for pH values ranging from 9.5 to 13, where the catholyte is depleted of divalent cations and CO_2_. The figure shows different extents of pH decrease (red–blue curves) with progressive CO_2_ absorption as pCO_2_ (gray curves) approaches −3.38 (i.e., atmospheric concentrations). For pCO_2_ evolution, increasing darkness of the gray curves corresponds to increasing initial pH of the catholyte effluent.

If uncompensated, i.e., by cation replenishment (for CaCO_3_) or by redissolution (for Mg(OH)_2_), the precipitation of Ca and Mg minerals from seawater (i.e., resulting in the removal of aqueous Ca^2+^ and Mg^2+^ species) in the catholyte would lead to a net lower seawater pH and hence a reduction in its dissolved CO_2_ storage capacity, as a function of CO_2_’s pH-dependent solubility in water (Figure 3a). Similarly, the decrease of the pH of the anolyte in an electrolysis system to pH ≈ 1 results in CO_2_’s degassing to a limit of 2.141 mmol CO_2_/kg seawater as described by Henry’s law (Figure 1a). But on the other hand, the net increase in the pH of the catholyte, on account of the electrolytic pH pump, and the subsequent dissolution of brucite, increases the amount of CO_2_ absorbed very significantly (Figure 1a), far exceeding the amount of CO_2_ degassed at the anolyte. For example, maintaining a fixed catholyte pH of 8.5, 9.0, and 9.5, just within the electrolytic reactor and not including the OH^–^ liberated following the dissolution of brucite, yields an *additional* 2.787, 17.72, and 86.54 mmol CO_2_/kg water of storage vis-à-vis the native pH of seawater (∼8.1) [N.B.: under operational conditions, the electrochemical reactors maintain a pH of 10–12 in the vicinity of the cathode]. This indicates that the *Equatic* process can enhance seawater’s intrinsic CO_2_ storage capacity, while also accomplishing atmospheric CO_2_ removal. This is in contrast to traditional direct air capture (DAC) processes since a decrease in atmospheric CO_2_ concentrations, if effected in isolation via DAC, would in fact, in time, result in the degassing of CO_2_ from the oceans on account of the ocean-atmosphere partitioning/exchange equilibrium of CO_2_ (eq 1).^1,41^

Expectedly, if the catholyte effluent is not in equilibrium with atmospheric CO_2_, re-equilibration, i.e., the progressive absorption of CO_2_ from the air, will decrease its pH (Figure 3b). Thus, our simulations show that an exit (effluent) pH ≈ 11.5 is required to maintain a pH ≥ 8.5 upon equilibration with atmospheric CO_2_, for a catholyte effluent that is depleted in aqueous Ca^2+^ and Mg^2+^ ions (i.e., where Ca and Mg are contained within mineral solids). It is furthermore important to highlight that, in the *Equatic* process, due to the provisioning of a continuous (electrolytic) pH pump, the precipitation of mineral carbonates does not result in the degassing of CO_2_ (i.e., due to acidification that results from the deprotonation of bicarbonate ions: HCO_3_^–^, during carbonate mineralization), as is the case for nonelectrolytically stimulated conditions.

#### Realkalinization of the Catholyte and Anolyte Effluent

**Figure 4:**
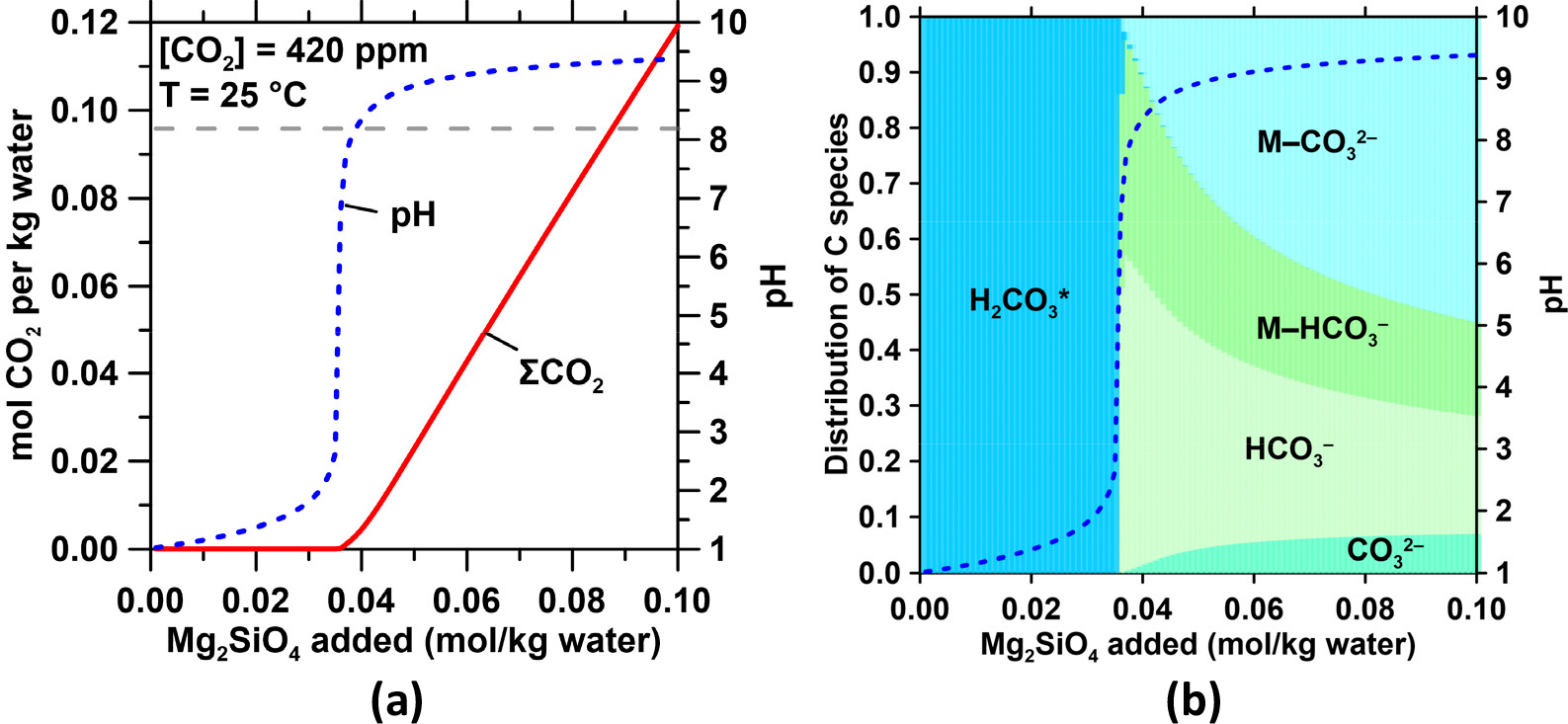
(a) The total dissolved inorganic carbon (ΣCO_2_) in the anolyte following dissolution (alkalinization) of Ca- or Mg-rich solids (e.g., Ca_2_SiO_4_ or Mg_2_SiO_4_). The dashed gray line indicates typical oceanic pH. (b) The distribution of inorganic carbon species as a function of the extent of realkalinization, showing the persistence of H_2_CO_3_* at low(er) pH and HCO_3_^–^ and CO_3_^2–^ at high(er) pH. M–HCO_3_^–^ and M–CO_3_^2–^ represent aqueous HCO_3_^–^ and CO_3_^2–^ complexes formed with Na^+^, Ca^2+^, and Mg^2+^ cations in solution.

The uncontrolled discharge of the anolyte (i.e., an acidic solution) effluent into the ocean could result in changes in seawater chemistry and saturation states (e.g., a decrease in SI with respect to aragonite, a reduced CO_2_ storage capacity, etc., Table 2, Table 3). To counter such effects requires the realkalinization of the effluent by the dissolution of alkaline minerals such as those found in mafic and ultramafic rocks into the anolyte, to elevate the concentrations of divalent cations. Candidate solutes for this include pyroxenes (e.g., augite: (Ca,Na)(Mg,Fe,Al,Ti)(Si,Al)_2_O_6_, diopside: MgCaSi_2_O_6_]) and olivines (e.g., forsterite: Mg_2_SiO_4_]) that naturally occur in mafic (basalts, gabbro) and ultramafic (peridotites) rocks. As Ca^2+^ and Mg^2+^ species are dissolved into the effluent, its pH and total dissolved CO_2_ content elevate (Figure 4). It is evident that an increase in the ΣCO_2_ occurs only when the pH exceeds ∼5 (Figure 1a). Furthermore, the replenishment of the cations increases not only the pH but also the salinity, enhancing CO_2_ absorption (Figures 1 & 4b,c)—i.e., a reason why seawater contains much more dissolved CO_2_ than freshwater.

**Figure 5:**
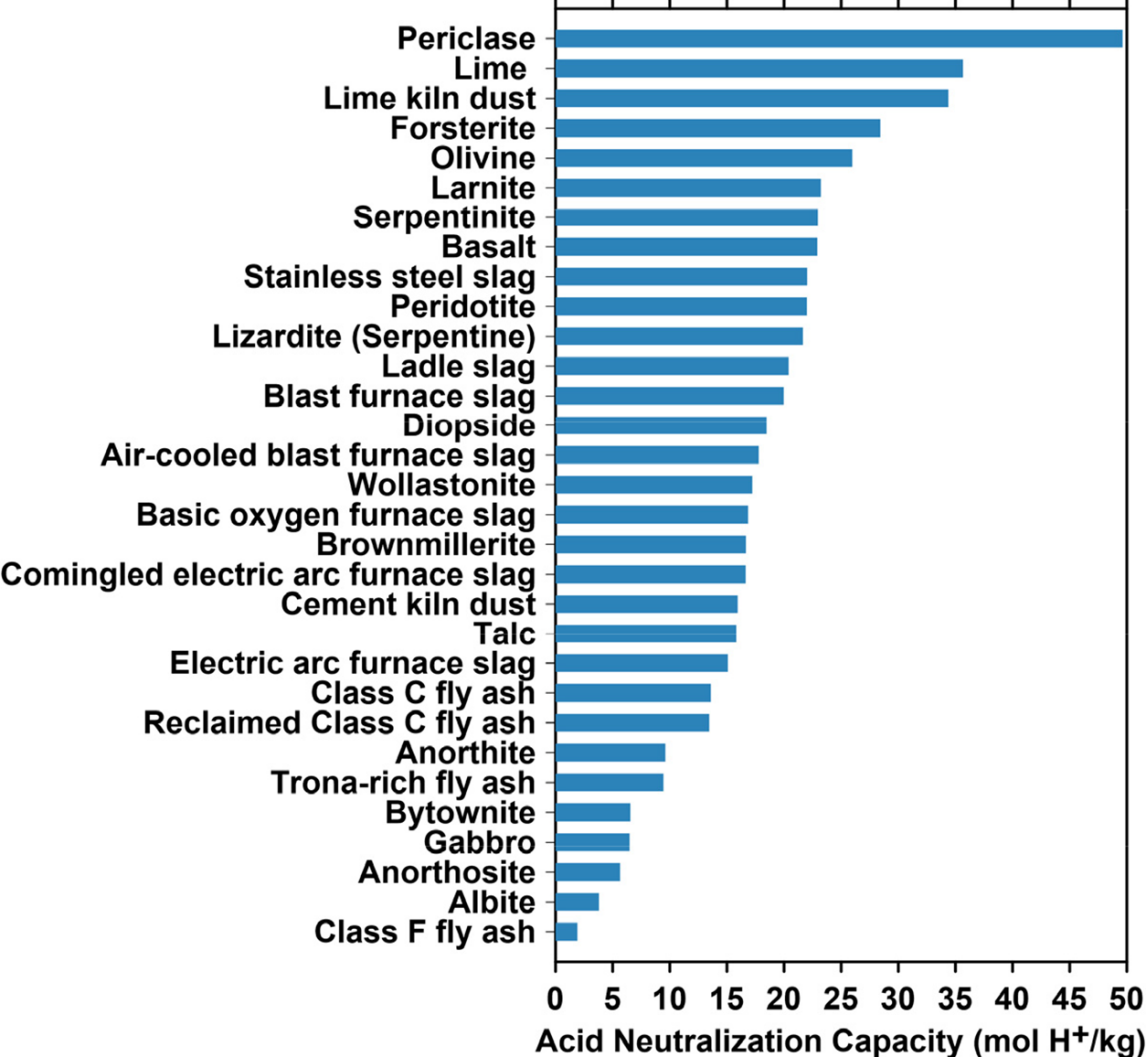
Acid neutralization capacity (ANC: i.e., the effluent realkalinization capacity) of diverse alkaline solids (Table 4). While exact abundances are nontrivial to assess, these materials are available at levels ranging from 10s-to-100s of millions (e.g., slags) to 1000s of billions of tonnes (e.g., olivine).

The quantity of rock required to enhance the cation abundance and the pH of the anolyte effluent is a function of the solute’s acid (H^+^, proton) neutralization capacity (ANC). This capacity can be calculated from a candidate solute/solute mixture’s oxide composition, assuming progressive dissolution, and a dissolution reaction (congruent: stoichiometric (e.g., Mg_2_SiO_4(s)_ (forsterite) + 4H^+^ → 2Mg^2+^ + H_4_SiO_4_) or incongruent: nonstoichiometric (e.g., CaAl_2_Si_2_O_8(s)_ (anorthite) + 2H^+^ + H_2_O → Ca^2+^ + Al_2_Si_2_O_5_(OH)_4(s)_)).^7^ For simplicity we consider complete and congruent dissolution (Table 4, Figure 5) to identify the maximum ANC. A range of compositions for these minerals yields ANCs of up to ∼50 mol H^+^/kg solid (i.e., for MgO). This translates to a theoretical mass (and volume) requirement of 1.60 g Mg_2_SiO_4_/g CO_2_ sequestered (0.49 cm^3^ Mg_2_SiO_4_/g CO_2_) or 2.36 g CaAl_2_Si_2_O_8_/g CO_2_ (0.86 cm^3^ CaAl_2_Si_2_O_8_/g CO_2_) to replenish Mg^2+^ or Ca^2+^ removed by precipitation of Mg–CO_3_ hydrates and CaCO_3_ (**Case 2b**). For **Cases 1** and **2a**, since the dissolution of Mg(OH)_2_ autogenously replenishes Mg^2+^ in seawater, only Ca^2+^ depletion needs to be considered, resulting in a solid requirement of 0.76 g CaAl_2_Si_2_O_8_/g CO_2_ (0.28 cm^3^ CaAl_2_Si_2_O_8_/g CO_2_). However, to additionally neutralize the acidity of the anolyte (i.e., OH^–^ from Mg(OH)_2_ dissolution is counted toward CO_2_ sequestration and thus cannot be double counted for acidity neutralization), an additional quantity of 1.07 g Mg_2_SiO_4_/g CO_2_ (0.45 cm^3^ Mg_2_SiO_4_/g CO_2_) is required.

**Table 4:** Diversity of Alkaline Solids That Can Be Used for Anolyte Re-Alkalinization Ordered as a Function of Their Stoichiometric Acid Neutralization Capacity (ANC)

**Solute**	**Description**	**ANC (mol H**^**+**^**/kg solid)**
Periclase	MgO, a mineral found in metamorphic rocks	49.63
Lime	CaO, can be naturally occurring or synthetic	35.66
Lime kiln dust^51^	Byproduct of lime manufacturing	34.38
Forsterite	Mg_2_SiO_4_, the Mg-endmember of olivine	28.43
Olivine^52^	Group of nesosilicate minerals found in ultramafic rocks	25.97
Larnite	Ca_2_SiO_4_, a nesosilicate found in crystalline slags	23.22
Serpentinite	Ultramafic rock rich in serpentine, a hydrothermal alteration product of olivine	22.96
Basalt	Fine-grained mafic rock rich in plagioclase feldspar and pyroxene	22.91
Stainless steel slag	Semicrystalline byproduct of metal manufacturing	22.02
Peridotite	Ultramafic rock rich in olivine with some pyroxene	22.00
Lizardite (Serpentine)	Mg_3_(Si_2_O_5_)(OH)_4_, a phyllosilicate	21.65
Ladle slag	Semicrystalline byproduct of metal manufacturing	20.40
Blast furnace slag^53^	Semicrystalline byproduct of metal manufacturing	19.97
Diopside	CaMgSi_2_O_6_, a single-chained inosilicate (pyroxene)	18.47
Air-cooled blast furnace slag	Crystalline byproduct of metal manufacturing	17.78
Wollastonite	CaSiO_3_, a single-chained inosilicate	17.22
Basic oxygen furnace slag	Semicrystalline byproduct of metal manufacturing	16.85
Brownmillerite	Ca_2_(Al,Fe^3+^)_2_O_5_, a nonstoichiometric perovskite	16.66
Comingled electric arc furnace slag	Semicrystalline byproduct of metal manufacturing	16.64
Cement kiln dust^53^	Amorphous byproduct of Ordinary Portland Cement (OPC) production	15.94
Talc	Mg_3_Si_4_O_10_(OH)_2_, a phyllosilicate	15.82
Electric arc furnace slag	Semicrystalline byproduct of metal manufacturing	15.08
Class C fly ash	High-calcium fly ash from processing subbituminous and lignite coals	13.59
Reclaimed Class C fly ash	High-calcium fly ash reclaimed from landfill	13.45
Anorthite	CaAl_2_Si_2_O_8_, Ca-endmember of plagioclase feldspar, a tectosilicate	9.61
Trona-rich fly ash	Fly ash containing Na_3_(CO_3_)(HCO_3_)·2H_2_O	9.44
Bytownite	Na_0.2_Ca_0.8_Al_1.8_Si_2.2_O_8_, a type of plagioclase feldspar, a solid solution of NaAlSi_3_O_8_ and CaAl_2_Si_2_O_8_	6.55
Gabbro	Coarse-grained mafic rock rich in plagioclase feldspar and pyroxene	6.48
Anorthosite	Fine-grained mafic rock rich in anorthite	5.65
Albite	NaAlSi_3_O_8_, Na-endmember of plagioclase feldspar, a tectosilicate	3.80
Class F fly ash	Low-calcium fly ash from processing anthracite and bituminous coals	1.91

The lower alkalinity requirement for **Cases 1** and **2a** is a result of the greater CO_2_ removal efficiency of Mg(OH)_2_ compared with Mg–CO_3_ hydrates (i.e., since only 1.2 mol of OH^–^ are required per mole of CO_2_ stored as dissolved bicarbonate (HCO_3_^–^) ions). For either case, if the catholyte effluent, i.e., including the suspended solids, were to be discharged into the ocean, the CaCO_3_ solids that are present would remain stable, i.e., they would not dissolve given the significant oversaturation of the oceans with respect to this mineral (see Table 2). That said, we recognize that effluent alkalinization (i.e., ensuring equivalence of the pH of the influent and the combined, anolyte + catholyte effluent) and divalent cation regeneration (i.e., abundances of Ca and Mg in the influent and combined effluent are equal) are prerequisite to discharge into the ocean. But, beyond chemical parameters, other aspects require further consideration. For example, it is known that the [Ca]/[Mg] ratio in the oceans is of relevance to calcifying organisms, particularly the stability of their calcified exoskeletons in an acidifying ocean.^42−45^ While we cannot yet assess if the *Equatic* process, if globally deployed for 10s of gigatonnes of CDR annually, would affect such aspects (albeit, not at the scale of a single or a few plants), further work is needed to better understand these details in due course.

Olivine ((Mg,Fe^2+^)_2_SiO_4_) is the most abundant mineral (ultramafic, and otherwise) in the Earth’s upper mantle. On the Earth’s surface, olivine is primarily found in ophiolites, which are sections of the uppermost mantle and oceanic crust that are exposed on land by tectonic activity and that are found worldwide along convergent and divergent plate boundaries. Ophiolites are composed of a specific sequence of mafic (basalt, gabbro) and ultramafic (peridotites such as harzburgite, dunite) rocks and can have thicknesses on the order of 5 to 10 km and encompass areas exceeding ∼100 000 km^2^.^46,47^ Peridotites are intrusive rocks that are classified based on the amounts of olivine, clinopyroxene ((Ca,Na,Li)(Mg,Fe^2+^,Al,Fe^3+^)Si_2_O_6_), and orthopyroxene ((Mg,Fe)Si_2_O_6_). In ophiolites, lherzolite, harzburgite, and dunite peridotites are most common, containing at least 40 mass % olivine. Assuming a thickness of 1 km, an area of 10 000 km^2^, and 50 mass % fosteritic olivine (Mg_2_SiO_4_) leads to a volume of 5000 km^3^, substantially exceeding the volume of olivine needed to sequester all anthropogenic CO_2_ emitted into the atmosphere, i.e., including ongoing (17 km^3^ per year; ∼36 Gt per year) and all legacy emissions (1225 km^3^; ∼2500 Gt since 1850).^48^ In other words, if appropriately harvested, natural mafic and ultramafic rocks and minerals are an effectively limitless supply of alkalinity for CO_2_ management. Beyond such rocks, industrial processes produce ∼7 Gt of alkaline solids per year, including (but not restricted to) products and byproducts such as lime (∼430 Mt), cement kiln dust (∼478 Mt), slags (∼516 Mt), and coal ash (∼701 Mt).^49,50^

**Figure 6:**
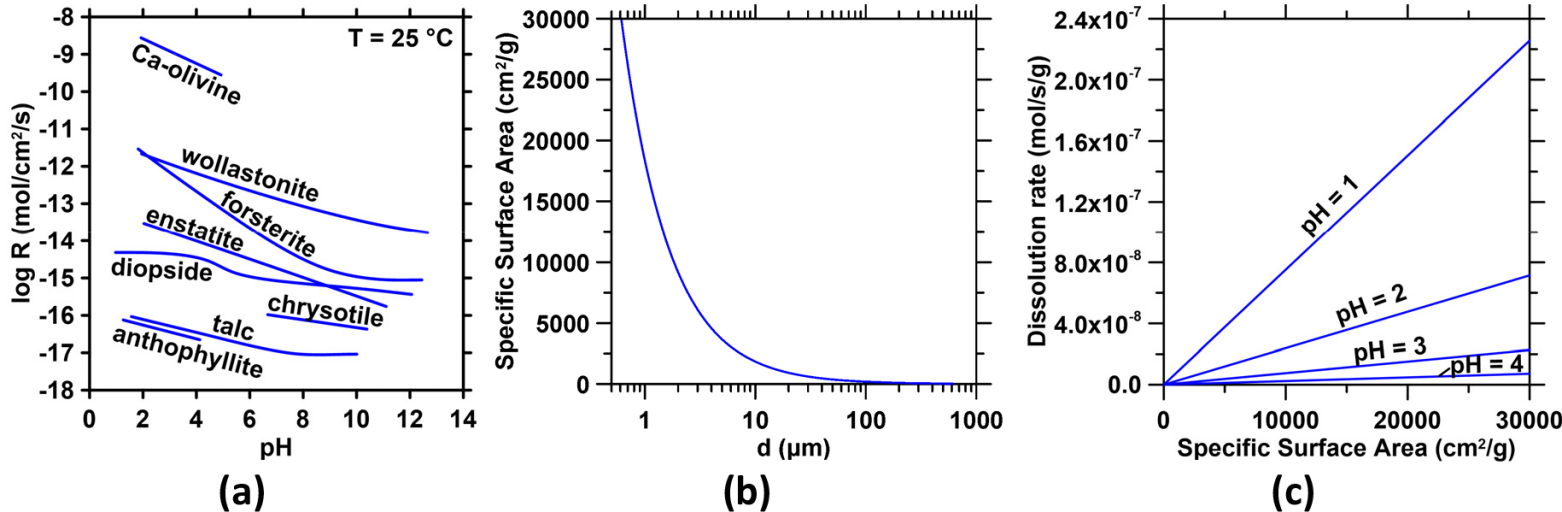
(a) The dissolution rate of Ca and Mg silicates at 25 °C as a function of pH, following Schott et al. (2009).^55^ (b) The dependence of the specific surface area on the diameter, assuming monosized spheres. (c) The mass-normalized dissolution rate of forsterite as a function of the specific surface area for select anolyte pH values, calculated from (a) and (b).

The Goldich stability series indicates that the relative reactivity of silicate minerals is dependent on their crystallization temperatures, i.e., minerals formed at higher temperatures are more reactive than those formed at lower temperatures (Figure 6a). This is further reflected in the degree of polymerization of tetrahedral silicate units, i.e., in general, a higher Si to O ratio signifies greater polymerization and lower temperature of cooling. For example, comparing the reactivities of anorthite and forsterite, both abundant silicates that are reservoirs of Ca and Mg, reveals that forsterite is more reactive owing to its structure in which all SiO_4_^4–^ units are connected to each other by Mg^2+^ ions.^54,55^ On the other hand, anorthite features a framework silicate structure with extensive sharing of O atoms by Si.^56^

In addition, kinetic considerations such as the rates of water exchange around the cations in an atomic structure also influence reactivity; for instance, Ca-based silicates dissolve faster than Mg-based silicates despite having an *equivalent* (crystallographic) structure due to the stronger solvation-state of Mg as compared to Ca.^57^ Using data from Pokrovsky and Schott (2000),^54^ the rate of forsterite dissolution as a function of pH for 1 < pH < 7 can be expressed as *R* = 2.376 × 10^–11^ e^–1.15(pH)^, where *R* is the dissolution rate in mol/cm^2^/s (see Figure 6a). Using this relation, the dissolution rates at pH 1, 2, 3, 4 can be estimated as 7.53 × 10^–12^, 2.38 × 10^–12^, 7.54 × 10^–13^, and 2.39 × 10^–13^ mol/cm^2^/s, respectively. For a nonporous sphere, i.e., where the external surface area represents the reactive surface area, the specific surface area (SSA) and particle diameter (*d*) are related by *d* = 6/(SSA × ρ), where ρ is the density (Figure 6b). The effect of specific surface area on mass-normalized dissolution rates can be used to assess the fineness of particles and the residence time in a column reactor that are required to achieve a sufficient rate and extent of mineral dissolution in the highly acidic anolyte to ensure divalent cation abundance renewal and realkalization (Figure 6c). The rate equations can also be used to calculate time-dependent pH evolution during mineral dissolution. For example, progressively dissolving 50 kg of forsterite (*d*_50_ = 10 μm) in 1000 kg of water (initial pH = 1) results in the release of ∼60 mol Mg^2+^ (60 mmol Mg^2+^/kg) within 24 h. For comparison, ∼80 mmol Mg^2+^/kg is required to raise the pH of the anolyte from ∼1 to ∼8.2 (i.e., native seawater pH) (Figure 4a). Decreasing the particle size increases the dissolution rate, at an energy expense. For instance, using a Bond Work Index (BWI) approach, for silicate rocks, we estimate that a grinding energy of around 70 MJ per tonne of rock (0.02 MWh/tonne) is needed to produce particles with *d*_50_ ≈ 100 μm for dissolution. Significantly, the intense acidity (pH ≈ 1) of the anolyte effluent that is generated herein is useful in that it enables accelerated silicate dissolution.

#### Dissolution of Brucite

**Figure 7:**
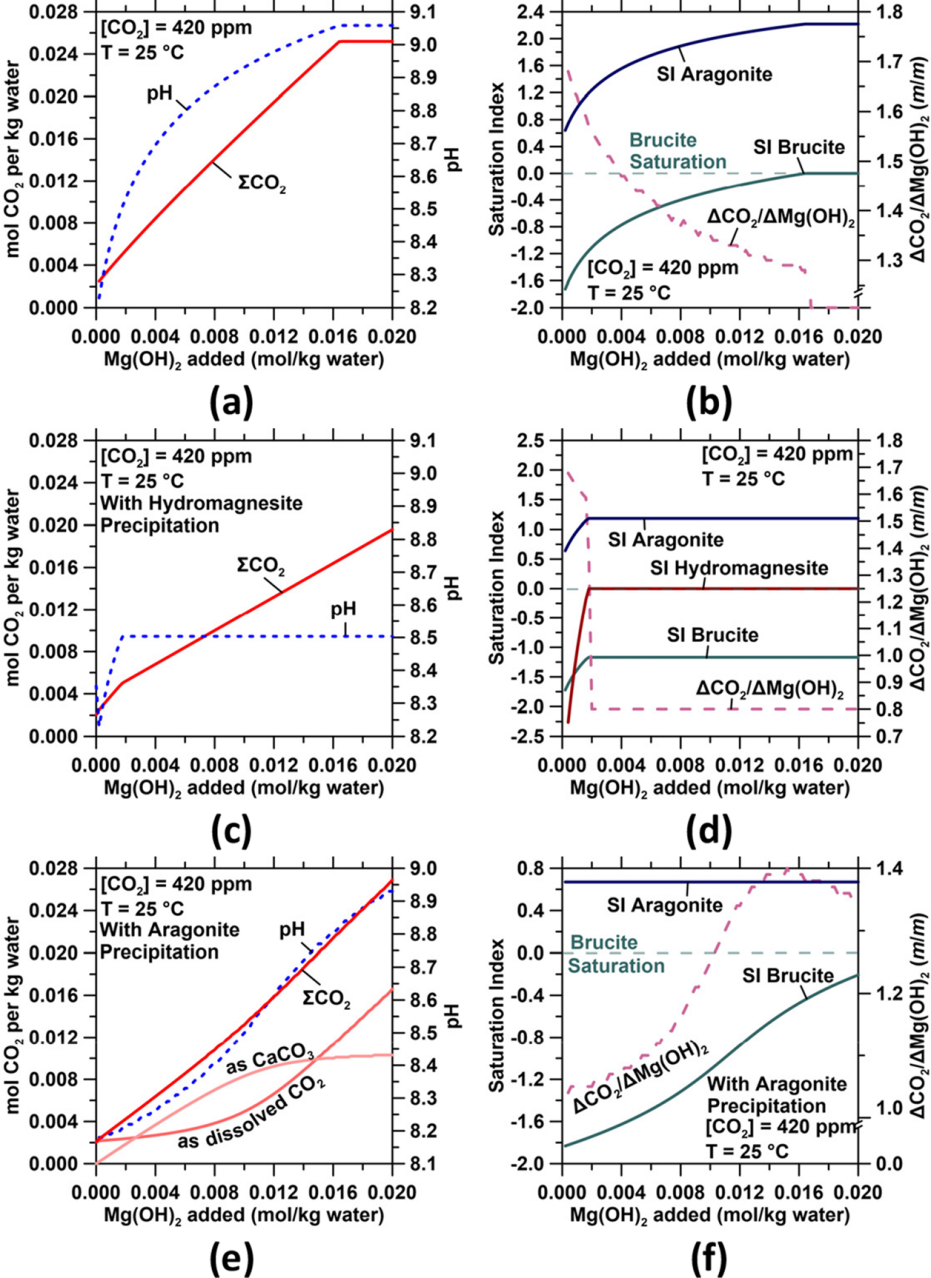
Effect of Mg(OH)_2_ addition/dissolution on the (a) total dissolved carbon and pH and (b) saturation indices of seawater with respect to aragonite and brucite. The molar effectiveness of Mg(OH)_2_ addition for CO_2_ removal for each 0.0002 mol of addition of Mg(OH)_2_ is also shown. (c) and (d) show plots similar to (a) and (b) for a case where hydromagnesite precipitation occurs when supersaturation is reached. The CO_2_ removal factor reduces to 0.8 mol of CO_2_ per mol of hydromagnesite (1 mol CO_2_ per mol nesquehonite). Similar plots as (a) and (b) for a case where aragonite precipitation occurs in excess of the original saturation index of seawater are shown in (e) and (f).

A range of diverse processes including riverine input, atmospheric and evaporite cycling, ion exchange, hydrothermal activity, low-temperature basalt weathering, and carbonate deposition control the input–output balance of divalent seawater ions (Ca^2+^, Mg^2+^) and the net change in seawater’s alkalinity.^58^ Natural processes result in a net flux for Ca that is zero (i.e., the amount added to the oceans is equal to the amount removed from the oceans by carbonate deposition) and a net decrease in Mg concentration by 1.5 × 10^12^ mol per year.^58^**Cases 1** and **2a** implies the dissolution of brucite in seawater, in which seawater is undersaturated, raising seawater’s pH while drawing down atmospheric CO_2_ thereby ensuring net CDR. The dissolution of brucite increases seawater’s pH expanding its CO_2_ storage capacity (Figure 7a) such that between 1.3-to-1.7 mol of CO_2_ are absorbed per mol of Mg(OH)_2_, assuming that no Mg–CO_3_ hydrates form (Figure 7b). The precipitation of Mg–CO_3_ hydrates (e.g., hydromagnesite: Mg_5_(CO_3_)_4_(OH)_2_·4H_2_O or nesquehonite: MgCO_3_·3H_2_O) under nondilute mass conditions within the plant reduces this value to 0.8-to-1.0 mol of CO_2_ removed per mol of Mg(OH)_2_ (Figure 7c,d). However, additional CO_2_ is absorbed if the Mg–CO_3_ hydrate solids are released into the ocean and progressively dissolve.

**Figure 8:**
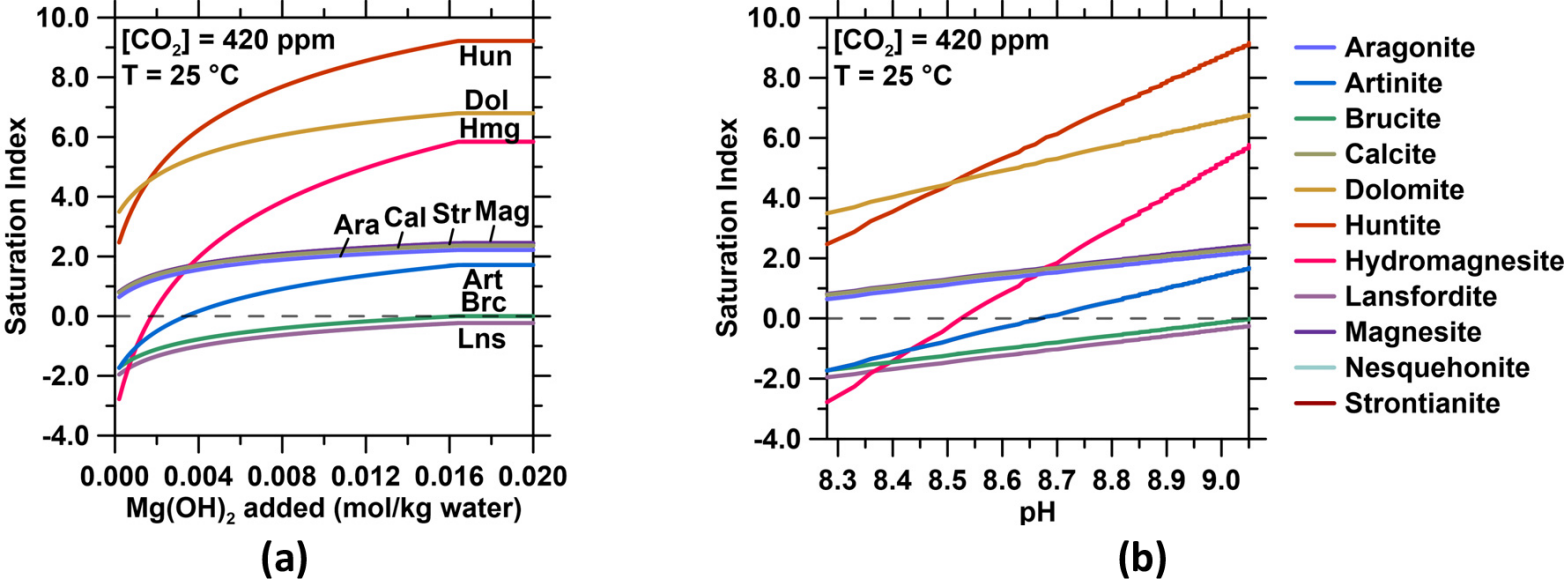
Changes in the mineral saturation indices of diverse minerals with (a) increasing Mg(OH)_2_ dissolution or (b) increasing pH, see also Table 2.

Under static/batch conditions (i.e., *R*_e_ → 0, where *R*_e_ is the Reynolds number, unitless) where there is very slow solutal transport, locally, it is possible that increases in the alkalinity of seawater, e.g., caused due to brucite dissolution, could alter mineral saturation states. This could induce secondary carbonate formation (see Figure 8a,b) since the increase in alkalinity shifts the HCO_3_^–^–CO_3_^2–^ distributions (e.g., see the Bjerrum diagram for dissolved inorganic carbon’s speciation) in seawater thereby resulting in aragonite precipitation via the combination of Ca^2+^ and CO_3_^2–^ species.^59^ Thus, in circumstances wherein the brucite-containing effluent is discharged into the oceans it is necessary to examine how quickly: (a) brucite may dissolve? and (b) how quickly dissolved brucite’s species (i.e., particularly alkalinity) may be transported? While the release of CO_2_ that typically accompanies secondary carbonate precipitation is not an issue (i.e., where the release of CO_2_ occurs via bicarbonate rather than carbonate combination with calcium species via the reaction: Ca^2+^ + 2HCO_3_^–^ → CaCO_3_ + CO_2_ + H_2_O, such that 1 mol of CO_2_ is released per mol of CaCO_3_ precipitated)^60^ in the presence of added alkalinity (i.e., Mg(OH)_2_ dissolution), this aspect should be considered in further detail because: (a) CaCO_3_ precipitation will consume OH^–^, decreasing the efficiency of CO_2_ absorption (Figure 7b–f) since it is more efficient to stabilize atmospheric CO_2_ as aqueous species than within mineral carbonates and (b) it could change locally and at short time scales the [Ca]/[Mg] ratios in seawater.

At seawater pH, the dissolution rate of brucite is on the order of 10^–8^ mol/m^2^/s.^61^ To achieve CO_2_ removal at the level of 10 Gt/year would require the dissolution of 8.3 Gt of Mg(OH)_2_ corresponding to the addition of 1.1 × 10^–7^ mol Mg(OH)_2_/kg seawater. If brucite’s dispersion is assumed to be averaged across the world’s oceans, an unrealistic and impractical assumption, changes in seawater pH and mineral saturation indices are irrelevant. For context, the critical saturation ratio, Ω, for runaway aragonite precipitation is reported to be 5 (SI = 0.69).^62^ Using a model that considers ocean circulation, i.e., ECCO (Estimating the Circulation and Climate of the Ocean) LLC270 physical fields,^63^ and constraints of ΔpH = 0.1 and ΔΩ_aragonite_ = 0.5, regions within 300 km of the coast can accommodate 100s of megatons of atmospheric CO_2_ removal.^64^ This shows that simple near-coastal alkalinity discharge, such as that proposed herein, can scale to several Gt per year of CDR if spread over available coastlines.^64^

But, an important question related to the *Equatic* process involves answering the question: *“What is the best approach for Mg(OH)_2_dissolution and atmospheric CO*_*2*_*drawdown: i.e., in the open ocean (**Case 1**), or within an (industrial) plant (**Cases 2a**,**2b**)?”* Each approach has distinct benefits and challenges. First, we can consider the case wherein brucite’s dissolution occurs following the discharge of the calcite and brucite (particulate) containing effluent into the ocean (“in ocean” approach). This requires two steps leading to CO_2_ removal: **Step A)** brucite dissolution, and **Step B)** CO_2_ drawdown from the atmosphere. At high brucite undersaturations and moderate convective conditions (e.g., turbulence in oceans varies by at least 8 orders of magnitude with characteristic *R*_e_ reported to range between 70 and 4 × 10^8^),^65−67^ the dissolution of brucite is rapid, requiring on the order of a few to 10s of hours. On the other hand, the equilibration of air and sea (i.e., gas–liquid CO_2_ concentrations) occurs over weeks to months depending on the mixed layer depth and wind speed.^68^ Therefore, to achieve atmospheric CO_2_ drawdown, Mg(OH)_2_ must not only fully dissolve but also the alkalinized seawater must remain in the mixed layer during this period. This results in an uncertainty regarding the amount of time and the extent of CO_2_ absorbed required for carbon dioxide drawdown from the atmosphere.

**Figure 9:**
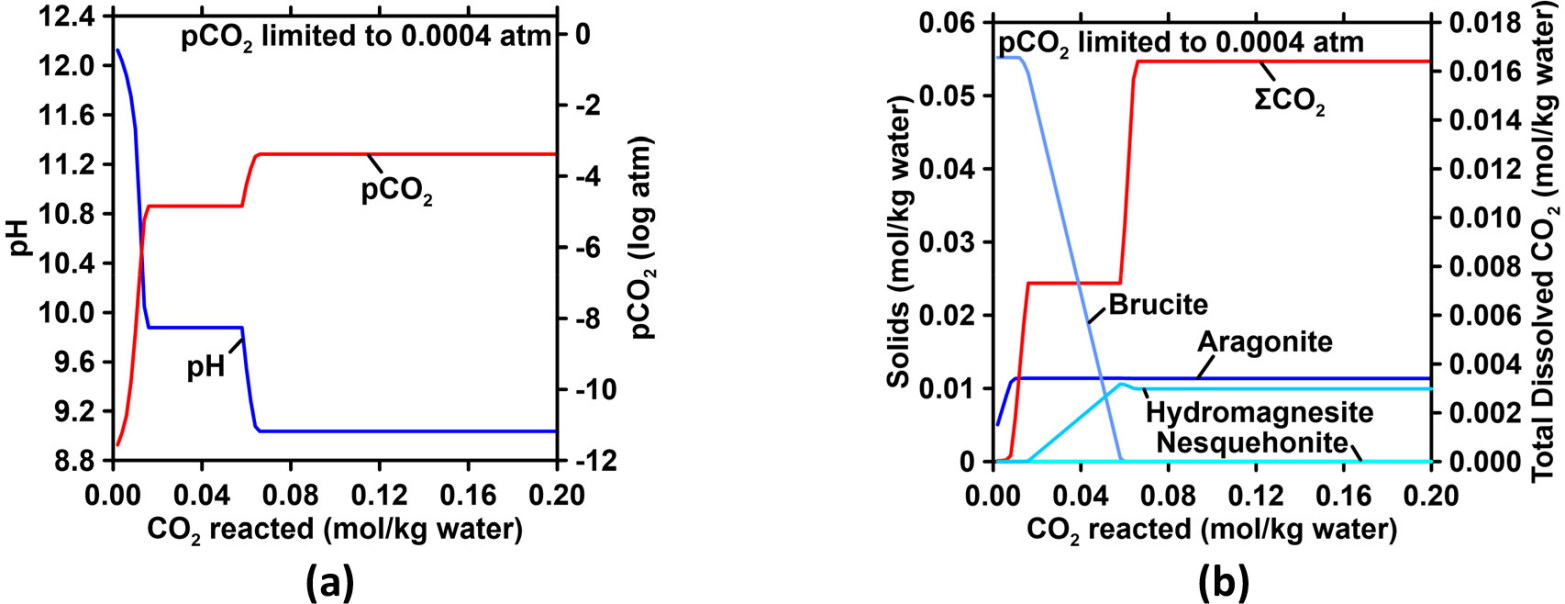
Changes in (a) pH, (b) solid phase assemblage, and total dissolved CO_2_ in the catholyte during reaction with CO_2_ to achieve equilibrium pCO_2_ equivalent to atmospheric conditions at 25 °C. These simulations show that the catholyte solids discharged include hydromagnesite and aragonite, in general agreement with our experiments.

Second, we can consider a process configuration wherein air is sparged into the brucite-containing catholyte within a high surface-to-volume (s/v, m^–1^), high mass transfer rate aeration reactor, i.e., inside-the-battery limit (“ISBL” approach) of an industrial plant. While such aeration requires bubbling ∼2500 t of atmospheric air to derive ∼1 t of CO_2_ (assuming ∼420 ppm of CO_2_ in air) the absorption of CO_2_ into the catholyte that contains CaCO_3_ and Mg(OH)_2_ results in progressive Mg(OH)_2_ dissolution and the immobilization of atmospherically derived CO_2_ in the form of HCO_3_^–^ and CO_3_^2–^ species, while the CaCO_3_ that is present remains unaffected. Careful analysis shows that, herein, all residual Ca^2+^ in solution in fact precipitates as CaCO_3_, while the dissolution of Mg(OH)_2_ in the presence of CO_2_ at high solid loadings results in the precipitation of hydromagnesite and nesquehonite, since magnesite formation is unachievable at ambient conditions (see Figure 9a,b). This allows for direct quantification of CO_2_ absorption as solid carbonates. Once released into the ocean, the hydrated magnesium carbonates redissolve, increasing alkalinity. This analysis matches our experimental observations, and as a result of progressive CO_2_ dissolution and stabilization, the pH of the catholyte decreases from ∼12.1 to ∼9.0, corresponding with the dissolution of brucite. The formation of Mg–CO_3_ hydrates can be avoided by employing low Mg(OH)_2_ solid loadings in the aeration reaction, resulting in greater CDR efficiency. While this ISBL approach requires aeration that implies an energy demand it is desirable in that it eliminates the uncertainty of CO_2_ removal and allows “direct and unambiguous, in plant” quantifications of both the rate and extent of CO_2_ removal. The obvious disadvantage is that it implies moving large quantities of air, which enhances the overall energy need of the process.

### Equatic’s Measurement, Reporting, and Verification (MRV) Approach for CO_2_ Removal

The net extent of CDR accomplished by the *Equatic* process must be measurable, verifiable, reportable, additional, and durable (permanent). In addition, the potential for leakage, harm, and cobenefits must be considered. Using the analysis presented in the sections above and in alignment with a recent approach suggested by CarbonPlan,^69^ we can calculate the net extent of CO_2_ removal effected by the *Equatic* process as follows:
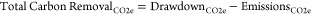
where, Emissions _CO2e_ includes the total embodied CO_2_ emissions from material and energy use (e.g., the grid emissions factor of electricity, and the amount of energy embodied in the coproduced hydrogen assuming typical purification demands, and conversion efficiencies), and
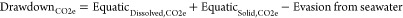
Using **Case 1** as an example, the CO_2_ sequestered as dissolved HCO_3_^–^ and CO_3_^2–^ ions and solid carbonates can be quantified unambiguously by weighing the masses of Mg(OH)_2_ and CaCO_3_ produced and multiplying these masses by a *carbon removal factor*, as follows (in units of g CO_2_ per m^3^ of water processed).
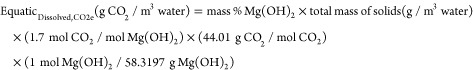

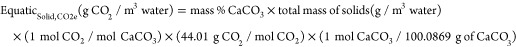


The total mass of the solids can be measured by separating the solids from the catholyte effluent stream, and the mass percentages of Mg(OH)_2_ and CaCO_3_ quantified—online, and in real-time, using thermogravimetric analysis. The mass percentages of Mg(OH)_2_ and CaCO_3_ are taken from the mass loss between 300-to-500 °C and 600-to-900 °C, respectively. The carbon removal factor for Equatic _Dissolved, CO2e_ is affected by the extent of Mg(OH)_2_ dissolution and the extent of CO_2_ absorption (into water) from air. The ISBL approach discussed above eliminates these uncertainties. The evasion of CO_2_ from seawater may result from secondary CaCO_3_ precipitation or the mixing of un-neutralized acid (anolyte), especially in the case of the “in ocean” approach, considerations of which are addressed above. While there are uncertainties regarding increasing the dissolved inorganic carbon (DIC) content of the oceans, notably, the *Equatic* approach counteracts ocean acidification that poses a significant risk to ocean ecosystems via a multitude of ways.^70,71^

## Summary and Conclusions

This paper presents a rigorous analysis of *Equatic*, an ocean-mediated process for CDR. We examine two limiting pathways for CDR, one in which CO_2_ is trapped solely within calcium and magnesium carbonates, and another in which CO_2_ is stored both as solid carbonates and as aqueous HCO_3_^–^ and CO_3_^2–^ by means of ocean alkalinity enhancement promoted by Mg(OH)_2_ dissolution. We carefully examine how the anolyte and catholyte effluents of the process present unique opportunities for rock dissolution and durable and permanent CO_2_ immobilization. We furthermore show how the process offers flexibility to eliminate the uncertainties associated with quantifying the rate and extent of CDR and minimize any detrimental changes in seawater composition and chemistry from the influent to the effluent. Furthermore, detailed considerations for realkalinization of the effluent including acid neutralization capacity and reactivity of diverse mineral solutes are discussed. This analysis provides the fundamental basis that justifies the viability of the approach and lays the foundation of a quantitative approach for MRV of the *Equatic* process.

## References

[ref1] La Plante E. C.; Simonetti D. A.; Wang J.; Al-Turki A.; Chen X.; Jassby D.; Sant G. N. Saline Water-Based Mineralization Pathway for Gigatonne-Scale CO_2_ Management. ACS Sustain. Chem. Eng. 2021, 9 (3), 1073–1089. 10.1021/acssuschemeng.0c08561.

[ref2] National Academies of Sciences, Engineering, and Medicine. Negative Emissions Technologies and Reliable Sequestration: A Research Agenda*;* The National Academies Press: Washington, DC, 2019. 10.17226/25259. 31120708

[ref3] Kelemen P. B.; McQueen N.; Wilcox J.; Renforth P.; Dipple G.; Vankeuren A. P. Engineered Carbon Mineralization in Ultramafic Rocks for CO_2_ Removal from Air: Review and New Insights. Chem. Geol. 2020, 550, 119628 10.1016/j.chemgeo.2020.119628.

[ref4] Matter J. M.; Stute M.; Snæbjornsdottir S. O.; Oelkers E. H.; Gislason S. R.; Aradottir E. S.; Sigfusson B.; Gunnarsson I.; Sigurdardottir H.; Gunnlaugsson E.; Axelsson G.; Alfredsson H. A.; Wolff-Boenisch D.; Mesfin K.; Taya D. F. d. l. R.; Hall J.; Dideriksen K.; Broecker W. S. Rapid Carbon Mineralization for Permanent Disposal of Anthropogenic Carbon Dioxide Emissions. Science 2016, 352 (6291), 1312–1314. 10.1126/science.aad8132. 27284192

[ref5] Mustafa J.; Mourad A. A.-H. I.; Al-Marzouqi A. H.; El-Naas M. H. Simultaneous Treatment of Reject Brine and Capture of Carbon Dioxide: A Comprehensive Review. Desalination 2020, 483, 114386 10.1016/j.desal.2020.114386.

[ref6] Ho H.-J.; Iizuka A. Mineral Carbonation Using Seawater for CO_2_ Sequestration and Utilization: A Review. Sep. Purif. Technol. 2023, 307, 122855 10.1016/j.seppur.2022.122855.

[ref7] La Plante E. C.; Mehdipour I.; Shortt I.; Yang K.; Simonetti D.; Bauchy M.; Sant G. N. Controls on CO_2_ Mineralization Using Natural and Industrial Alkaline Solids under Ambient Conditions. ACS Sustain. Chem. Eng. 2021, 9 (32), 10727–10739. 10.1021/acssuschemeng.1c00838.

[ref8] Veizer J.; Hoefs J.; Lowe D. R.; Thurston P. C. Geochemistry of Precambrian Carbonates: II. Archean Greenstone Belts and Archean Sea Water. Geochim. Cosmochim. Acta 1989, 53 (4), 859–871. 10.1016/0016-7037(89)90031-8. 11539784

[ref9] Sundquist E. T. Geological Perspectives on Carbon Dioxide and the Carbon Cycle. In The Carbon Cycle and Atmospheric CO_2_: Natural Variations Archean to Present*;* American Geophysical Union (AGU), 1985; pp 55–59. 10.1029/GM032p0005.

[ref10] Renforth P.; Henderson G. Assessing Ocean Alkalinity for Carbon Sequestration. Rev. Geophys. 2017, 55 (3), 636–674. 10.1002/2016RG000533.

[ref11] Caldeira K.; Akai M.. Ocean Storage. In IPCC special report on carbon dioxide capture and storage*;* Cambridge University Press: Cambridge, 2005.

[ref12] Watson A. J.; Schuster U.; Shutler J. D.; Holding T.; Ashton I. G. C.; Landschützer P.; Woolf D. K.; Goddijn-Murphy L. Revised Estimates of Ocean-Atmosphere CO_2_ Flux Are Consistent with Ocean Carbon Inventory. Nat. Commun. 2020, 11 (1), 4422 10.1038/s41467-020-18203-3. 32887875 PMC7474059

[ref13] House K. Z.; House C. H.; Schrag D. P.; Aziz M. J. Electrochemical Acceleration of Chemical Weathering as an Energetically Feasible Approach to Mitigating Anthropogenic Climate Change. Environ. Sci. Technol. 2007, 41 (24), 8464–8470. 10.1021/es0701816. 18200880

[ref14] Xie H.; Liu T.; Hou Z.; Wang Y.; Wang J.; Tang L.; Jiang W.; He Y. Using Electrochemical Process to Mineralize CO_2_ and Separate Ca^2+^/Mg^2+^ Ions from Hard Water to Produce High Value-Added Carbonates. Environ. Earth Sci. 2015, 73 (11), 6881–6890. 10.1007/s12665-015-4401-z.

[ref15] Xie H.; Liu T.; Wang Y.; Wu Y.; Wang F.; Tang L.; Jiang W.; Liang B. Enhancement of Electricity Generation in CO_2_ Mineralization Cell by Using Sodium Sulfate as the Reaction Medium. Appl. Energy 2017, 195, 991–999. 10.1016/j.apenergy.2017.03.072.

[ref16] Sharifian R.; Wagterveld R. M.; Digdaya I. A.; Xiang C.; Vermaas D. A. Electrochemical Carbon Dioxide Capture to Close the Carbon Cycle. Energy Environ. Sci. 2021, 14 (2), 781–814. 10.1039/D0EE03382K.

[ref17] Sharifian R.; Boer L.; Wagterveld R. M.; Vermaas D. A. Oceanic Carbon Capture through Electrochemically Induced in Situ Carbonate Mineralization Using Bipolar Membrane. Chem. Eng. J. 2022, 438, 135326 10.1016/j.cej.2022.135326.

[ref18] Intergovernmental Science-Policy Platform on Biodiversity and Ecosystem Services. Summary for Policymakers of the Methodological Assessment of the Diverse Values and Valuation of Nature of the Intergovernmental Science-Policy Platform on Biodiversity and Ecosystem Services (IPBES)*;* Zenodo: Bonn, Germany, 2022. 10.5281/zenodo.7410287.

[ref19] Parkhurst D. L.; Appelo C. A. J. Description of Input and Examples for PHREEQC Version 3—A Computer Program for Speciation, Batch-Reaction, One-Dimensional Transport, and Inverse Geochemical Calculations. In U.S. Geological Survey Technology Methods Book; USGS, 2013; book 6, chapter A43. Online at https://pubs.usgs.gov/tm/06/a43/

[ref20] Millero F. J.; Feistel R.; Wright D. G.; McDougall T. J. The Composition of Standard Seawater and the Definition of the Reference-Composition Salinity Scale. Deep Sea Res. Part Oceanogr. Res. Pap. 2008, 55 (1), 50–72. 10.1016/j.dsr.2007.10.001.

[ref21] NOAA US Department of Commerce. Global Monitoring Laboratory - Carbon Cycle Greenhouse Gases. https://gml.noaa.gov/ccgg/trends/mlo.html (accessed 2021-09-08).

[ref22] Dickson A. G. An Exact Definition of Total Alkalinity and a Procedure for the Estimation of Alkalinity and Total Inorganic Carbon from Titration Data. Deep Sea Res. Part Oceanogr. Res. Pap. 1981, 28 (6), 609–623. 10.1016/0198-0149(81)90121-7.

[ref23] Wolf-Gladrow D. A.; Zeebe R. E.; Klaas C.; Körtzinger A.; Dickson A. G. Total Alkalinity: The Explicit Conservative Expression and Its Application to Biogeochemical Processes. Mar. Chem. 2007, 106 (1), 287–300. 10.1016/j.marchem.2007.01.006.

[ref24] Middelburg J. J.; Soetaert K.; Hagens M. Ocean Alkalinity, Buffering and Biogeochemical Processes. Rev. Geophys. 2020, 58 (3), e2019RG000681 10.1029/2019RG000681. PMC7391262 32879922

[ref25] Zeebe R. E.; Wolf-Gladrow D. CO2 in Seawater: Equilibrium, Kinetics, Isotopes*,* 3rd Impression 2005 (with corrections); Elsevier Science: Amsterdam; New York, 2001.

[ref26] Mook W. G. Chemistry of Carbonic Acid in Water. In Environmental Isotopes in the Hydrological Cycle: Principles and Applications*;* International Atomic Energy Agency, 2001; Vol. 1, pp 87–98.

[ref27] Dickson A. G. The Carbon Dioxide System in Seawater: Equilibrium Chemistry and Measurements. Guide Best Pract. Ocean Acidif. Res. Data Report. 2010, 1, 17–40.

[ref28] Millero F. J.; Roy R. N. A Chemical Equilibrium Model for the Carbonate System in Natural Waters. Croat. Chem. Acta 1997, 70 (1), 1–38.

[ref29] Dickson A. G.; Millero F. J. A Comparison of the Equilibrium Constants for the Dissociation of Carbonic Acid in Seawater Media. Deep Sea Res. Part Oceanogr. Res. Pap. 1987, 34 (10), 1733–1743. 10.1016/0198-0149(87)90021-5.

[ref30] Orr J. C.; Epitalon J.-M. Improved Routines to Model the Ocean Carbonate System: Mocsy 2.0. Geosci. Model Dev. 2015, 8 (3), 485–499. 10.5194/gmd-8-485-2015.

[ref31] Wanninkhof R.; Lewis E.; Feely R. A.; Millero F. J. The Optimal Carbonate Dissociation Constants for Determining Surface Water pCO_2_ from Alkalinity and Total Inorganic Carbon. Mar. Chem. 1999, 65 (3), 291–301. 10.1016/S0304-4203(99)00021-3.

[ref32] Butler J. N. Ionic Equilibrium: Solubility and pH Calculations*;* John Wiley & Sons: Sudbury, MA, 1998.

[ref33] Hain M. P.; Sigman D. M.; Higgins J. A.; Haug G. H. The Effects of Secular Calcium and Magnesium Concentration Changes on the Thermodynamics of Seawater Acid/Base Chemistry: Implications for Eocene and Cretaceous Ocean Carbon Chemistry and Buffering. Glob. Biogeochem. Cycles 2015, 29 (5), 517–533. 10.1002/2014GB004986.

[ref34] Shaojun Z.; Mucci A. Calcite Precipitation in Seawater Using a Constant Addition Technique: A New Overall Reaction Kinetic Expression. Geochim. Cosmochim. Acta 1993, 57 (7), 1409–1417. 10.1016/0016-7037(93)90002-E.

[ref35] Sabbides T.; Giannimaras E.; Koutsoukos P. G. The Precipitation of Calcium Carbonate in Artificial Seawater at Sustained Supersaturation. Environ. Technol. 1992, 13 (1), 73–80. 10.1080/09593339209385130.

[ref36] Lin Y.-P.; Singer P. C.; Aiken G. R. Inhibition of Calcite Precipitation by Natural Organic Material: Kinetics, Mechanism, and Thermodynamics. Environ. Sci. Technol. 2005, 39 (17), 6420–6428. 10.1021/es050470z. 16190195

[ref37] Zuddas P.; Mucci A. Kinetics of Calcite Precipitation from Seawater: II. The Influence of the Ionic Strength. Geochim. Cosmochim. Acta 1998, 62 (5), 757–766. 10.1016/S0016-7037(98)00026-X.

[ref38] Burton E. A.; Walter L. M. The Role of PH in Phosphate Inhibition of Calcite and Aragonite Precipitation Rates in Seawater. Geochim. Cosmochim. Acta 1990, 54 (3), 797–808. 10.1016/0016-7037(90)90374-T.

[ref39] Bischoff J. L. Catalysis, Inhibition, and the Calcite-Aragonite Problem; [Part] 2, The Vaterite-Aragonite Transformation. Am. J. Sci. 1968, 266 (2), 80–90. 10.2475/ajs.266.2.80.

[ref40] Mucci A.; Canuel R.; Zhong S. The Solubility of Calcite and Aragonite in Sulfate-Free Seawater and the Seeded Growth Kinetics and Composition of the Precipitates at 25°C. Chem. Geol. 1989, 74 (3), 309–320. 10.1016/0009-2541(89)90040-5.

[ref41] Cao L.; Caldeira K. Atmospheric Carbon Dioxide Removal: Long-Term Consequences and Commitment. Environ. Res. Lett. 2010, 5 (2), 024011 10.1088/1748-9326/5/2/024011.

[ref42] Cohen A. L.; McCorkle D. C.; de Putron S.; Gaetani G. A.; Rose K. A. Morphological and Compositional Changes in the Skeletons of New Coral Recruits Reared in Acidified Seawater: Insights into the Biomineralization Response to Ocean Acidification. Geochem. Geophys. Geosystems 2009, 10 ( (7), ). 10.1029/2009GC002411.

[ref43] Fabry V. J.; Seibel B. A.; Feely R. A.; Orr J. C. Impacts of Ocean Acidification on Marine Fauna and Ecosystem Processes. ICES J. Mar. Sci. 2008, 65 (3), 414–432. 10.1093/icesjms/fsn048.

[ref44] Milazzo M.; Rodolfo-Metalpa R.; Chan V. B. S.; Fine M.; Alessi C.; Thiyagarajan V.; Hall-Spencer J. M.; Chemello R. Ocean Acidification Impairs Vermetid Reef Recruitment. Sci. Rep. 2014, 4 (1), 4189 10.1038/srep04189. 24577050 PMC5379440

[ref45] Segev E.; Erez J. Effect of Mg/Ca Ratio in Seawater on Shell Composition in Shallow Benthic Foraminifera. Geochem. Geophys. Geosystems 2006, 7 ( (2), ). 10.1029/2005GC000969.

[ref46] Dilek Y. Ophiolite Concept and Its Evolution. Geol. Soc. Am. 2003. 10.1130/0-8137-2373-6.1.

[ref47] Ali M. Y.; Watts A. B.; Searle M. P.; Keats B.; Pilia S.; Ambrose T. Geophysical Imaging of Ophiolite Structure in the United Arab Emirates. Nat. Commun. 2020, 11 (1), 2671 10.1038/s41467-020-16521-0. 32471992 PMC7260221

[ref48] Schuiling R. D.; De Boer P. L. Coastal Spreading of Olivine to Control Atmospheric CO_2_ Concentrations: A Critical Analysis of Viability. Comment: Nature and Laboratory Models Are Different. Int. J. Greenh. Gas Control 2010, 4 (5), 855 10.1016/j.ijggc.2010.04.012.

[ref49] Statista. Global production of lime 2021. Statista. https://www.statista.com/statistics/1006040/production-of-lime-worldwide/ (accessed 2022-07-25).

[ref50] Renforth P. The Negative Emission Potential of Alkaline Materials. Nat. Commun. 2019, 10 (1), 1401 10.1038/s41467-019-09475-5. 30923316 PMC6438983

[ref51] Latif M. A.; Naganathan S.; Razak H. A.; Mustapha K. N. Performance of Lime Kiln Dust as Cementitious Material. Procedia Eng. 2015, 125, 780–787. 10.1016/j.proeng.2015.11.135.

[ref52] Ward’s® Olivine (Fine). VWR. https://www.wardsci.com/store/product/8882507/ward-s-olivine-fine (accessed 2022-05-10).

[ref53] Kalina L.; Bílek V.; Kiripolský T.; Novotný R.; MáSilko J. Cement Kiln By-Pass Dust: An Effective Alkaline Activator for Pozzolanic Materials. Mater. Basel Switz. 2018, 11 (9), E1770 10.3390/ma11091770. PMC6164556 30235786

[ref54] Pokrovsky O. S.; Schott J. Kinetics and Mechanism of Forsterite Dissolution at 25°C and pH from 1 to 12. Geochim. Cosmochim. Acta 2000, 64 (19), 3313–3325. 10.1016/S0016-7037(00)00434-8.

[ref55] Schott J.; Pokrovsky O. S.; Oelkers E. H. The Link Between Mineral Dissolution/Precipitation Kinetics and Solution Chemistry. Rev. Mineral. Geochem. 2009, 70 (1), 207–258. 10.2138/rmg.2009.70.6.

[ref56] Oelkers E. H.; Schott J. Experimental Study of Anorthite Dissolution and the Relative Mechanism of Feldspar Hydrolysis. Geochim. Cosmochim. Acta 1995, 59 (24), 5039–5053. 10.1016/0016-7037(95)00326-6.

[ref57] Brantley S. L. Kinetics of Mineral Dissolution. In Kinetics of Water-Rock Interaction*;* Brantley S. L., Kubicki J. D., White A. F., Eds.; Springer New York: New York, NY, 2008; pp 151–210. 10.1007/978-0-387-73563-4_5.

[ref58] McDuff R. E.; Morel F. M. M. The Geochemical Control of Seawater (Sillen Revisited). Environ. Sci. Technol. 1980, 14 (10), 1182–1186. 10.1021/es60170a007.

[ref59] Fuhr M.; Geilert S.; Schmidt M.; Liebetrau V.; Vogt C.; Ledwig B.; Wallmann K. Kinetics of Olivine Weathering in Seawater: An Experimental Study. Front. Clim. 2022, 4.

[ref60] Nguyen Dang D.; Gascoin S.; Zanibellato A.; G. Da Silva C.; Lemoine M.; Riffault B.; Sabot R.; Jeannin M.; Chateigner D.; Gil O. Role of Brucite Dissolution in Calcium Carbonate Precipitation from Artificial and Natural Seawaters. Cryst. Growth Des. 2017, 17 (4), 1502–1513. 10.1021/acs.cgd.6b01305.

[ref61] Pokrovsky O. S.; Schott J. Experimental Study of Brucite Dissolution and Precipitation in Aqueous Solutions: Surface Speciation and Chemical Affinity Control. Geochim. Cosmochim. Acta 2004, 68 (1), 31–45. 10.1016/S0016-7037(03)00238-2.

[ref62] Moras C. A.; Bach L. T.; Cyronak T.; Joannes-Boyau R.; Schulz K. G. Ocean Alkalinity Enhancement &ndash; Avoiding Runaway CaCO_3_ Precipitation during Quick and Hydrated Lime Dissolution. Biogeosciences Discuss. 2021, 1–31. 10.5194/bg-2021-330.

[ref63] Carroll D.; Menemenlis D.; Adkins J. F.; Bowman K. W.; Brix H.; Dutkiewicz S.; Fenty I.; Gierach M. M.; Hill C.; Jahn O.; Landschützer P.; Lauderdale J. M.; Liu J.; Manizza M.; Naviaux J. D.; Rödenbeck C.; Schimel D. S.; Van der Stocken T.; Zhang H. The ECCO-Darwin Data-Assimilative Global Ocean Biogeochemistry Model: Estimates of Seasonal to Multidecadal Surface Ocean pCO_2_ and Air-Sea CO_2_ Flux. J. Adv. Model. Earth Syst. 2020, 12 (10), e2019MS001888 10.1029/2019MS001888.

[ref64] He J.; Tyka M. D. Limits and CO_2_ Equilibration of Near-Coast Alkalinity Enhancement. EGUsphere Prepr. 2022, 1–26. 10.5194/egusphere-2022-683.

[ref65] Thorpe S. A. An Introduction to Ocean Turbulence*;* Cambridge University Press: New York, 2007.

[ref66] Barkley R. A. Johnston Atoll’s Wake. J. Mar. Res. 1972, 30 (2), 201–216.

[ref67] Moum J. N. Variations in Ocean Mixing from Seconds to Years. Annu. Rev. Mar Sci. 2021, 13, 201–226. 10.1146/annurev-marine-031920-122846. 32600217

[ref68] Jones D. C.; Ito T.; Takano Y.; Hsu W.-C. Spatial and Seasonal Variability of the Air-Sea Equilibration Timescale of Carbon Dioxide. Glob. Biogeochem. Cycles 2014, 28 (11), 1163–1178. 10.1002/2014GB004813.

[ref69] Chay F.; Klitzke J.; Hausfather Z.; Martin K.; Freeman J.; Cullenward D. Verification Confidence Levels for Carbon Dioxide Removal; CarbonPlan, 2022. https://carbonplan.org/research/cdr-verification-explainer.

[ref70] Doney S. C.; Busch D. S.; Cooley S. R.; Kroeker K. J. The Impacts of Ocean Acidification on Marine Ecosystems and Reliant Human Communities. Annu. Rev. Environ. Resour. 2020, 45 ( (1), ).

[ref71] Kroeker K. J.; Kordas R. L.; Crim R.; Hendriks I. E.; Ramajo L.; Singh G. S.; Duarte C. M.; Gattuso J.-P. Impacts of Ocean Acidification on Marine Organisms: Quantifying Sensitivities and Interaction with Warming. Glob. Change Biol. 2013, 19 (6), 1884–1896. 10.1111/gcb.12179. PMC3664023 23505245

